# Mortality amongst European children with congenital anomalies: Associations with socio-economic status in the EUROLINKCAT cohort

**DOI:** 10.1371/journal.pone.0352025

**Published:** 2026-08-05

**Authors:** Sue Jordan, David Tucker, Ieuan Scanlon, Daniel S. Thayer, Elisa Ballardini, Clara Cavero-Carbonell, Mads Damkjaer, Miriam Gatt, Mika Gissler, Lyubov Ostapchuk, Michel Santoro, Sarah Stevens, Diana Wellesley, Wladimir Wertelecki, Joanne Given, Maria Loane, Hywel T. Evans

**Affiliations:** 1 Faculty of Medicine, Health and Life Sciences, Swansea University, Swansea, United Kingdom; 2 Congenital Anomaly Register & Information Service for Wales (CARIS), Public Health Knowledge and Research, Public Health Wales, Swansea, United Kingdom; 3 Department of Medical Sciences, Neonatal Intensive Care Unit, University Hospital of Ferrara, IMER Registry (Emilia Romagna Registry of Birth Defects), University of Ferrara, Ferrara, Italy; 4 Rare Diseases Research Unit, Foundation for the Promotion of Health and Biomedical Research in the Valencian Region, Valencia, Spain; 5 Department of Paediatrics and Adolescent Medicine, Lillebaelt Hospital, University Hospital of Southern Denmark, Kolding, Denmark; 6 Malta Congenital Anomalies Registry, Directorate for Health Information and Research, Pieta, Malta; 7 Department of Data and Analytics, THL Finnish Institute for Health and Welfare, Helsinki, Finland; Region Stockholm, Academic Primary Health Care Centre, Stockholm, Sweden; 8 Department of Molecular Medicine and Surgery, Karolinska Institute, Stockholm, Sweden; 9 Rivne Medical Diagnostic Center and OMNI-Net Ukraine, Rivne Oblast, Ukraine; 10 Unit of Epidemiology of Rare Diseases and Congenital Anomalies, Institute of Clinical Physiology, National Research Council, Pisa, Italy; 11 National Disease Registration Service, NHS, London, England; 12 University of Southampton and Wessex Clinical Genetics Service, Princess Anne Hospital, Southampton, United Kingdom; 13 Institute of Nursing and Health Research, Ulster University, Belfast, United Kingdom; Canakkale Onsekiz Mart University School of Medicine, TÜRKIYE

## Abstract

**Background:**

Childhood mortality is affected by major congenital anomalies and socio-economic status (SES). To our knowledge, their combined impact has not been explored to age 10.

**Methods:**

We analysed the population-based EUROlinkCAT cohort to ascertain the impact of SES on mortality by ages 1 and 10 amongst children with congenital anomalies in ten congenital anomaly registers in seven European countries. Four countries defined SES according to maternal education, and two used their national index of multiple deprivation. The ten registers used a common script to generate data on survival by ages 1 and 1–10 (365–3651 days). Eight registers analysed deaths according to their SES criteria, categorised as low, intermediate and high SES. Linked data were combined in random effects meta-analyses. Finland and Emilia-Romagna held data on single motherhood and EU-nationality.

**Results:**

We analysed mortality in relation to SES using data on 47,134 live-born children with major congenital anomalies classified and recorded 1996–2014. Mortality by age 1 and ages 1–10 was higher amongst the most than the least deprived, hazard ratios (HR) and 95% confidence intervals (CI) 1.47, 1.19–1.83 and 2.00, 1.32–3.02. Differences between intermediate and least deprived groups were smaller. Differences were statistically significant for all four analyses only in Ukraine and Wales. Mortality rates were higher for children of non-EU nationals, but not single mothers.

**Implications:**

Deprivation was more strongly associated with death at ages 1–10 than in infancy. These analyses of the most ill children in Europe indicate that, to achieve sustainable development goals, more resources are needed for the most vulnerable children.

## Background

There has been little change in childhood mortality from amenable causes over recent decades [1], and sustainable development goals for child survival are not being met [2,3]. Congenital anomalies affected 2.13% (2.13–2.15%) live births in Europe 2005–2022, with little change in 20 years [4]. Children with congenital anomalies have higher mortality rates than those without [5], and socio-economic status (SES) affects survival in children throughout infancy [6], childhood [7], and into adulthood [8]. However, less is known about the children affected by both congenital anomalies and adverse social circumstances, outside the USA [9]. The impact of socio-economic status (SES) on prematurity and subsequent mortality [6], injury and infant mortality is substantial [10]. Death from childhood cancer [11] and congenital heart disease (CHD) of moderate severity [12] are associated with lower SES and rurality. However, to our knowledge, the association has not been explored beyond infancy [13,14] for children with congenital anomalies, other than CHD [15,16]. Survival of children with anomalies has been compared across countries [17], but it is important to review any differences within countries. To our knowledge, it is not known whether the impact of SES on infant and childhood mortality extends to survival by age 10 in children with congenital anomalies. The aim of this paper is to identify the impact of SES on children already vulnerable due to major congenital anomalies and describe any differences across Europe.

## Methods

### Study design

A data-linkage cohort derived from ten population-based EUROCAT registers in seven European countries was used to explore mortality amongst infants and children with major congenital anomalies born 1995–2014. Data on live births with any major congenital anomaly, defined according to EUROCAT criteria [18], were linked to population data to ascertain survival to ages one and 10, plus the SES of the mother at birth [19]. Mortality records were accessed to the child’s 10^th^ Birthday or 31.12.2015, whichever was earlier. Survival to ages 1 and 10 were analysed separately; this reflected transition from hospital into community care [20]. We report in accordance with the Reporting of Studies Conducted using Observational Routinely Collected Data (RECORD) guidelines (S1 RECORD Checklist in S1 File) [21].

### Settings

Eight congenital anomaly registers from six countries linked to whole-population data on survival (mortality data) and SES (maternal data). Two different proxy variables for SES were used: maternal education and maternal index of multiple deprivation (IMD). Each country reported data on only one SES measure. Two other registers (Finland and Emilia Romagna) held data on nationality and marital status. Information on the wealth and prosperity of the country or region, as GDP *per capita,* and income inequality, as GINI coefficient, was obtained from available open access public domain sources, to illustrate and contextualise the work, following precedents [22–25]. Population infant mortality, and SES gradations in infant and childhood mortality rates were reported to illustrate comparability with whole-population mortality (Table 1); some 20–31% infant and childhood deaths are attributed to congenital anomalies [26–28].

**Table 1:** Participating countries and regions, data available and National Statistics.

Register	SES measure	% infants linked to other data *	GDP per capita 2022†	GINI coefficient for income 2019*‡*	Infant mortality per 1,000 live births 2022 **	Infant mortality per 1,000 live births, population and SES***	Childhood mortality in population per 100,000 and SES***
Denmark, Funen	Maternal education	100	$74,005	0.28	3.3	Most deprived tertile 7.5, least deprived 5.1. Rate ratio 1.5	1-10 years, most deprived tertile 28, least deprived 18. Rate ratio 1.5, 1981–2000
Finland	Only marital status and nationality	99.9	$59,027	0.28	2.0	Most deprived tertile 2.80, least deprived 1.36. Rate ratio 2.1	1−10 years, most deprived tertile 10.8, least deprived 7.4. Rate ratio 1.5, 2020−24
Italy, Emilia Romagna	Only marital status and nationality	91.4	$51,865	0.35	2.3	No data available	No data available
Italy, Tuscany	Maternal education	87.2	$51,865	0.35	2.3	No data available	No data available
Malta	Maternal education	100% to mortality records	$55,928	0.31	5.3	No data available	No data available
Ukraine	Maternal education	100% deaths reported	$12,671	0.27	7.2	No data collected under martial law	No data collected under martial law
England, Thames Valley	Area based IMD	87.8	£39,921 / $51,018 SE England	0.33	3.0	All England: most deprived decile 6.1; least deprived 2.2. Rate ratio 2.8 [28,29]	All England, ages 0–17, note below.
England, EMSY	Area based IMD	89.5	£29,683/ $37,934 E midlands	0.33	4.3	See Thames Valley	See Thames Valley
England, Wessex	Area based IMD	86.4	S West £33,657 /$43,013	0.33	2.8	See Thames Valley	See Thames Valley
Wales	Area based WIMD	99.7	£27,274 / $34,856	0.30	3.6	Most deprived decile 7.2; least deprived 3.6. Rate ratio 2 [28]	1-10, most deprived quintile: 21.5, least deprived quintile: 13.3, rate ratio 1.6 [, 30]

Notes to Table 1. EMSY, East Midlands and South Yorkshire, GDP, gross domestic product, IMD, index of multiple deprivation, SE, south-east, SES, socio-economic status, UK, United Kingdom, WIMD, Welsh index of multiple deprivation

*Data from [19]

†GDP per capita 2022 for:

• Wales and England regions [31] Regional boundaries are not contiguous with registers’ boundaries, and approximations have been made. GBP were converted to USD at a rate of 1.28, November 2024. https://www.calculator.net/currency-calculator.html

•Other European registers [32] UK GDP per capita was $54,603. Congruity has been assumed.

‡GINI coefficient / index for income:

• Wales and UK regions [33] Regions of England differed in their GINI coefficients for wealth, with London being the highest, and the South West being the lowest. No data for GINI coefficients for income were located for English regions, and the value for England was substituted [34].

• Other European registers [35,36]

** Infant mortality: Wales and UK regions Office for National Statistics [37], Other European registers [38]

***Data were not available for English regions. All-England data are available for infant mortality according to SES. S1 Table in S2 File has further details, based on the limited data in the public domain. Rate ratios ranged 1.6 to 3.2. Childhood mortality is available for all-England for ages 0–17 for 2023 [39]. South East and South West England have lower mortality rates than other regions: the East Midlands’ rate is intermediate. Rates according to deprivation quintiles for all England are 48.1 (most deprived) and 18.7 (least deprived) deaths per 100,000 children, rate ratio 2.6. Due to geographical differences, these data are not comparable with those reported in this study.

We were unable to obtain data for Italy, Malta and Ukraine. EUROSTAT does not hold data on childhood mortality. EUROSTAT reports infant mortality by year, country, region, and causes, but data on infant mortality according to SES are restricted to selected countries and as numbers only [40].

For Finland, national data were extracted from the Invest Full Population Data study 2020−24 (https://invest.utu.fi/fi/investkokonaisdata/information-about-the-research/).

For Wales, national data were extracted from the Secure Anonymised Information Linkage (SAIL) Databank. The rate ratio for 2018 is 1.7 [26].

For Denmark, national data were extracted for 1981–2000 from [41]. Figures quoted are for boys; those for girls are similar.

### Socioeconomic status (SES)

Socioeconomic status (SES) describes an individual’s effective social situation, relative to the population. SES reflects determinants of the positions of individuals or groups within the structure of a society [42], and encompasses concepts with diverse theoretical, historical and disciplinary origins [43]. SES is a relative, not absolute, measure, specific to each register: the most disadvantaged of some countries may be better situated materially than the most advantaged of others. Quality of life and social capital, which can be leveraged for advantage, are aligned with SES [44,45]. Most epidemiological studies include a measure of SES. Variations include: income, time in education, occupation or address, which is used to calculate a deprivation score, rank, quintile or decile. Both area-based and individual measures of SES affect mortality [46]. Selection of SES measure depended on data availability, quality, and advice of register leads [47].

#### Indices of deprivation.

The four countries of the UK each have their own index of multiple deprivation (IMD) [48], which have superseded Townsend scores and ranks [49]. The English Index of Multiple Deprivation (IMD) has seven components: a) Income b) Employment c) Education, Skills and Training d) Health and Disability e) Crime f) Barriers to Housing and Services g) Living Environment, based on 39 indicators [50]. The Wales 2014 Index of Multiple Deprivation (WIMD) is a composite of eight domains (or types) of deprivation: a) Income b) Employment c) Health d) Education e) Access to Services f) Community Safety g) Physical Environment h) Housing. Each domain is compiled from several different indicators, and the domains are combined to give a single rank [51].

#### Education.

Maternal education data were grouped by the source registers based on EUROCAT guide 1.4 (p.34) [18], which is based on UNESCO’s Standard Classification of Education 1997 [52]. Given the low numbers in each register, the categories were reduced to ‘elementary or to end of compulsory schooling’, ‘secondary, usually to age 18’ and ‘tertiary’. No registers held data on paternal education.

For all eight registers, SES (proxy) measures were divided into three groups (Table 2). The 3 central quintiles of the UK IMD scores were combined.

**Table 2:** Socio-economic Status Coding.

Proxy variable for SES	Coding
Maternal education (as in EUROCAT 1.4) [18]:	• Tertiary/post-secondary = High • Any secondary = Middle • Primary/pre-primary/No education = Low
Multiple Deprivation Index at birth	• Quintile 1 (Least deprived) = High • Quintiles 2–4 = Middle • Quintile 5 (Most deprived) = Low

Finland and Emilia-Romagna recorded nationality, migration and marital status of mothers, but not SES. Nationality of mother and child were classified as: national of the country of the register, other EU national, non-EU national. Maternal marital status was described as single, long-term relationship or widowed.

### Linkage

Data from EUROCAT registers were linked with a) maternal data to obtain maternal SES, marital status or nationality, and b) administrative data to obtain data on mortality. Linkage was undertaken as described [19], and ranged from 86% (Wessex, England) to 100% (Funen, Denmark) (Table 1). Registers and birth years where >15% of records were not linked to mortality records were excluded. Infants with congenital anomalies who could not be linked with national data sets were more likely to have died within a week of birth [19]; they, like others with no linked information on SES, are excluded from this analysis.

### Outcome

We report on infant deaths (0–364 days of life) and deaths 1–10 years of age (365–3651 days) to live born children with major congenital anomalies. Date of death was obtained from official statistics, such as Office on National Statistics (ONS) register of births and deaths.

### Analysis

The main analytical approaches for this EUORLINKCAT study were published before data were accessed [47]. Variables were coded using a Common Data Model (CDM) [53,54]. Individual linked case data were analysed in each participating register using a common STATA syntax script.

We included all live-born children with major anomalies whose matches between the EUROCAT register and mortality data were rated as ‘excellent / good’ as assessed by register leads [19], and excluded those where the match was rated ‘fair/ poor’) (Table 1). Each centre sent results to the Central Results Repository *via* a secure portal. Results were collated for random effects meta-analyses. Univariable Cox proportional hazards models estimated the hazard ratios (HRs) of death, with 95% confidence intervals (CI), for the ‘most deprived’ SES and the intermediate categories compared with the most affluent at 1 and 10 years. Registers with non-convergence of the proportional hazards model (for example, due to insufficient numbers of events) were excluded from meta-analyses on a case-by-case basis. Missing data were not imputed. Heterogeneity was assessed using the I^2^ statistic. A P value of >0.1 was considered suggestive of clinical heterogeneity [55]. Cox proportional hazard ratios in individual congenital anomalies were reported for individual countries where >50 deaths occurred, without meta-analysis [54]. Data were visualised as scatter plots of the natural logarithm of the hazard ratios [56].

### Governance and Ethical approvals

All EUROCAT registers have ethical and information governance clearance plus permissions required under national guidelines for routine population surveillance, data collection and transmission of anonymised data to a central database. Permissions to link EUROCAT data to mortality or vital statistics and to transmit linked anonymous aggregate data and analytic results to a Central Results Repository for combination and meta-analysis were obtained by each centre, as described [19,57]. University of Ulster obtained ethics permission for the Central Results Repository on 15 September 2017 (Institute of Nursing and Health Research Ethics Filter Committee, number FCNUR-17–000).

### Patient and public involvement

EUROlinkCAT study priorities were informed by discussions with parents’ advisory groups which connected researchers with families across Europe living with congenital anomalies to involve them at the start of the study [58]. In all registers, patient and public representatives are involved in project approvals.

## Results

The number of records available for analysis varied with the size of the population served by the register, the prevalence of reported congenital anomalies, and the start and end dates of the register. In total, data on 95,584 live-born children with a major congenital anomaly were available for analysis. Across the ten EUROCAT registers with data available, the infant mortality rate for children with anomalies ranged from 7.9%, 7.0–9.0% in Malta to 2.9%, 2.4–3.4% in Tuscany. Nine registers held childhood mortality data to 10 years. In most countries, a further 1–2% of live born children died before their 10^th^ year; the exception was Ukraine, where an additional 9% children died. Mortality between ages 1–10 was highest in Ukraine 13.5%, 12.5–14.6%, and lowest in Tuscany, 3.7%, 3.1–4.4% (Table 3). The registers contributing the highest number of children, Finland and Wales, had lower mortality rates.

**Table 3:** Numbers of livebirths with a major congenital anomaly and risk of death.

Register	Included birth years	Total livebirths	Mean follow-up, years	Risk of death, birth to 364 days, % (95%CI)	Risk of death, birth to 10 years (3651 days), % (95%CI)
Denmark, Funen^a^	1995-2014	2,425	7.5	5.4 (4.6, 6.4)	6.2 (5.3, 7.3)
Finland	1995-2014	42,861	7.2	3.5 (3.3, 3.7)	4.3 (4.1, 4.5)
Italy, Emilia Romagna	2008-2014	5,589	4.1	3.3 (2.9, 3.8)	^b^
Italy, Tuscany	2005-2014	4,312	5.6	2.9 (2.4, 3.4)	3.7 (3.1, 4.4)
Malta^c,d^	1995-2014	2,718	7.4	7.9 (7.0, 9.0)	9.0 (8.0, 10.1)
Ukraine ^d^	2006-2014	5,836	4.2	4.3 (3.8, 4.9)	13.5 (12.5,14.6)
England, Thames Valley	2005-2013	3,854	5.1	6.7 (6.0, 7.6)	8.1 (7.3, 9.1)
England, EMSY	2003-2012	11,288	6.6	6.9 (6.4, 7.4)	8.4 (7.9, 9.0)
England, Wessex	2004-2014	4,360	5.6	6.5 (5.8, 7.3)	8.3 (7.4, 9.3)
Wales	1998-2014	18,177	7.0	3.8 (3.6, 4.1)	4.9 (4.6, 5.2)

Notes to Table 3. CI, confidence interval; EMSY, East Midlands and South Yorkshire.

^a^counts rounded to nearest 5 due to statistical disclosure control.

^b^not available.

^c^termination of pregnancy is illegal in Malta.

^d^linked to mortality databases only.

### Infant mortality and SES

**Fig 1:**
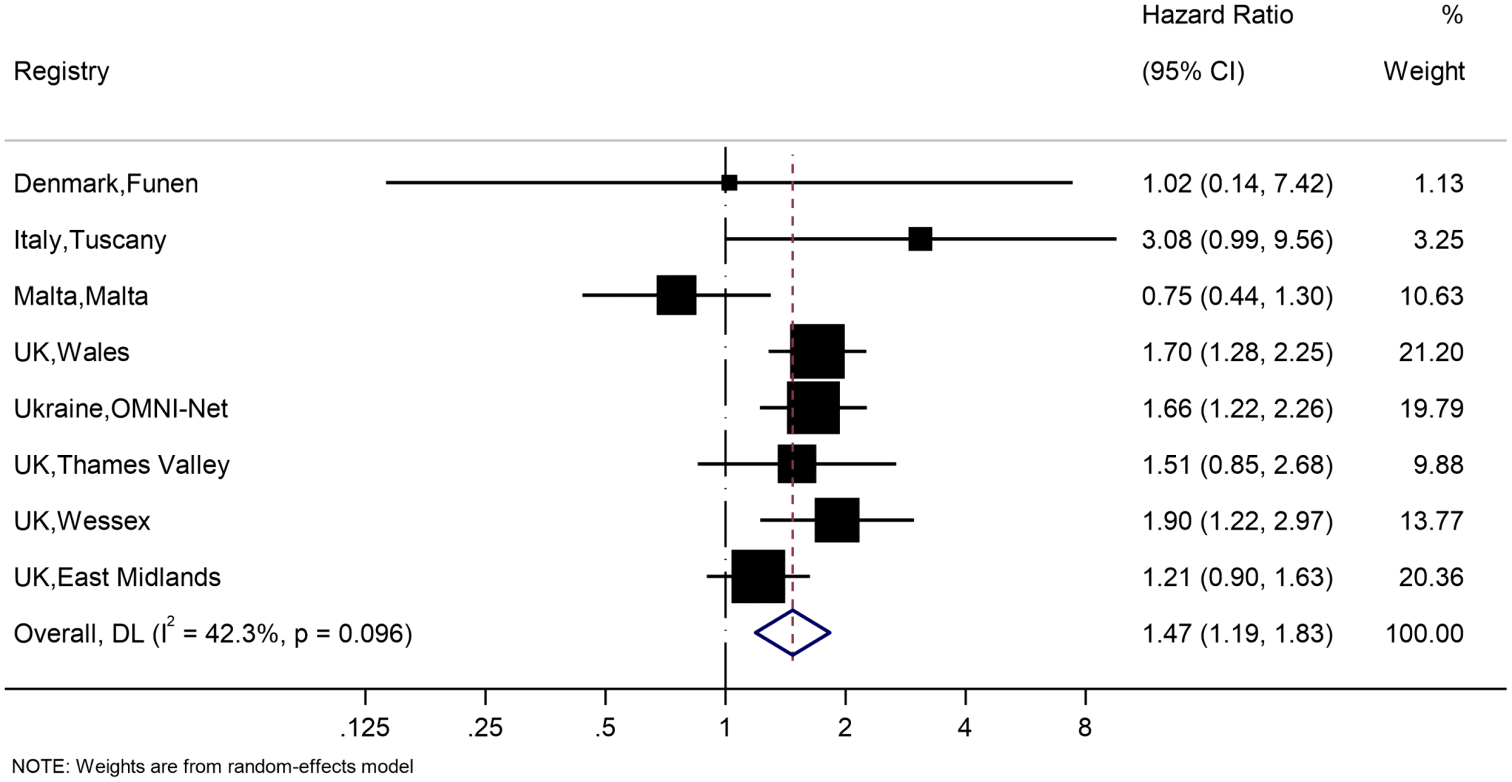
Hazard ratios for Infant Mortality for children with Lowest SES vs Highest (most affluent) SES.

**Fig 2:**
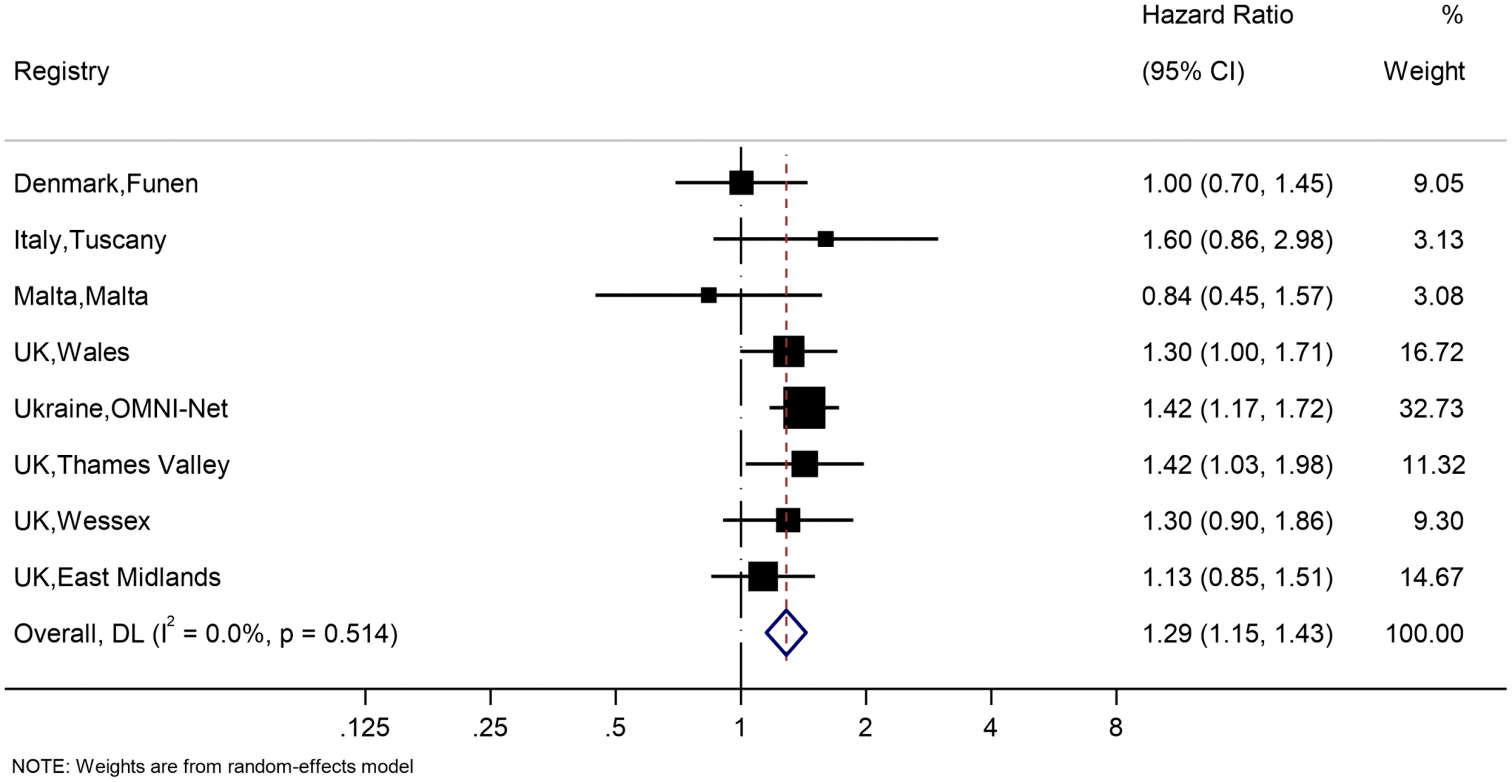
Hazard ratios for Infant Mortality for children with Middle SES vs Highest (most affluent) SES. Notes to Figs 1 and 2. CI confidence interval, DL DerSimonian and Laird, a standard method for fitting a random-effects model for meta-analysis.

Eight registers, including 3 from England, were able to link infant mortality with a measure of SES for 47,134 children. The impact of SES on infant mortality was more marked for the most deprived (Hazard ratio [HR] 1.47, 1.19–1.83) than the intermediate group (HR 1.29, 1.15–1.43). In Malta infant mortality was lower amongst those with lower SES, but the difference was not statistically significant. The greatest disparity was seen in Tuscany, but this did not reach statistical significance, due to low numbers of deaths. Statistically significant differences for both lowest and intermediate groups were seen in Ukraine and Wales. The Wessex and Thames Valley regions of England had significant disparities for the lowest and intermediate groups, respectively. Modest clinical heterogeneity was observed (Figs 1 and 2).

### Mortality ages 1–10 and SES

**Fig 3:**
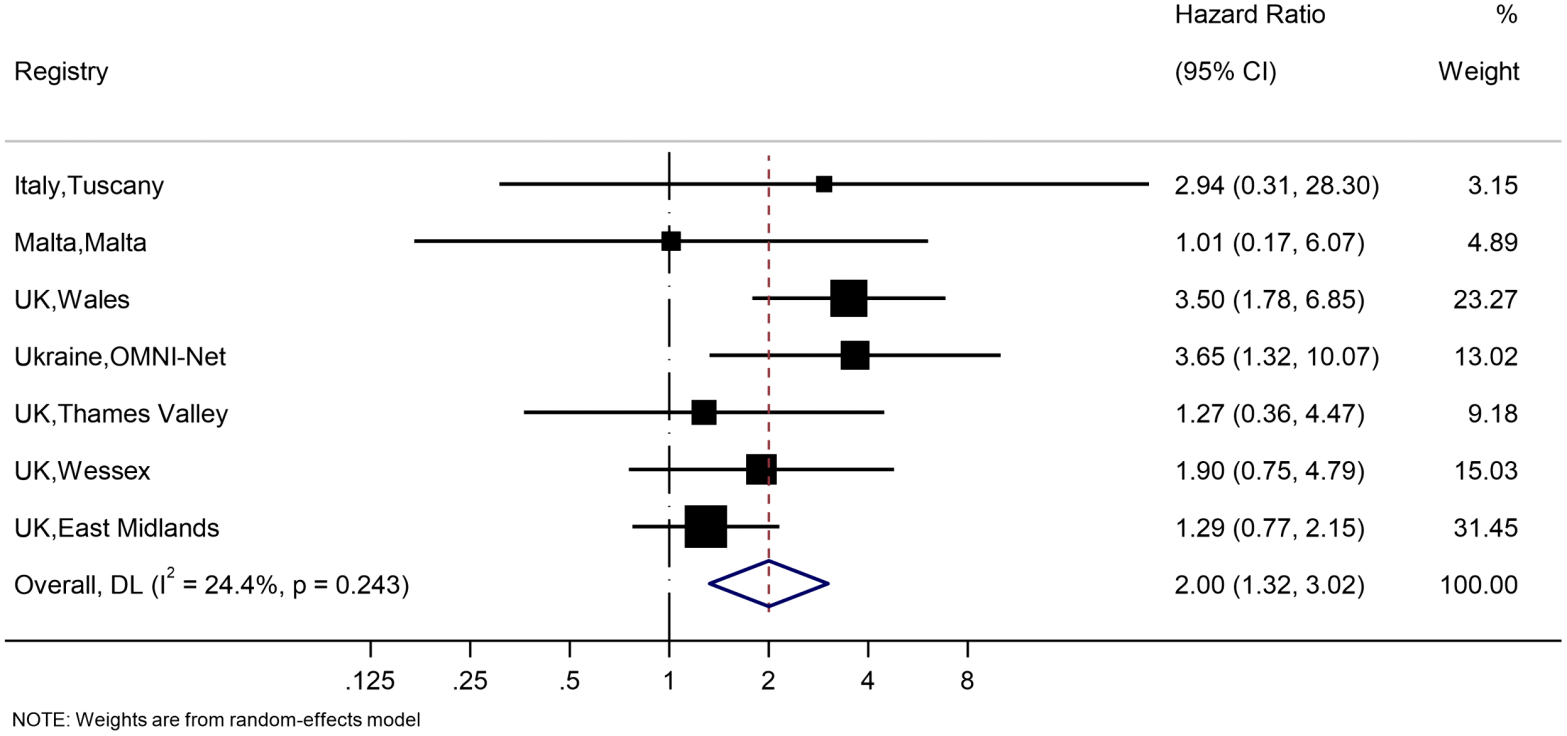
Hazard ratios for death in years 1 to 9 for children with Lowest vs Highest (most affluent) SES.

**Fig 4:**
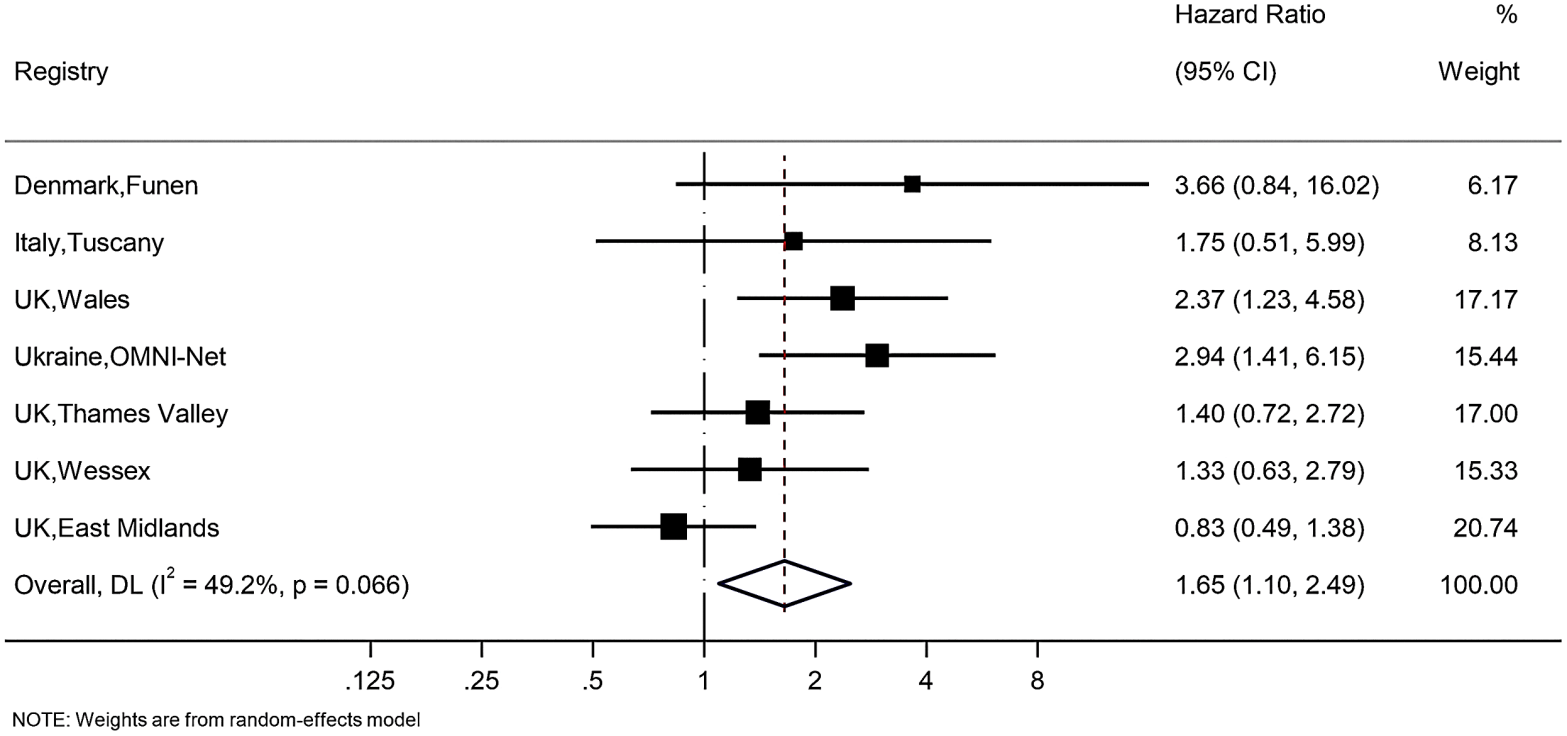
Hazard ratios for death in years 1 to 9 for children with Middle vs Highest (most affluent) SES. Notes to Figs 3 and 4. CI confidence interval, DL DerSimonian and Laird, a standard method for fitting a random-effects model for meta-analysis.

The low numbers of deaths in this age group precluded reporting in Funen, Denmark (too few deaths in the high deprivation category), leaving 7 registers. SES was a stronger determinant of survival between ages 1–10 than in the first year of life. Overall, the impact of SES on mortality was more marked for the most deprived (HR 2.00, 1.32–3.02) than the intermediate group (HR 1.65, 1.10–2.49). Statistically significant associations were seen in Wales and Ukraine. SES had no impact in Malta, and the middle quintiles appeared to be protected in the English East Midland (EMSY) region. Modest clinical heterogeneity was observed (Figs 3, 4).

**Fig 5:**
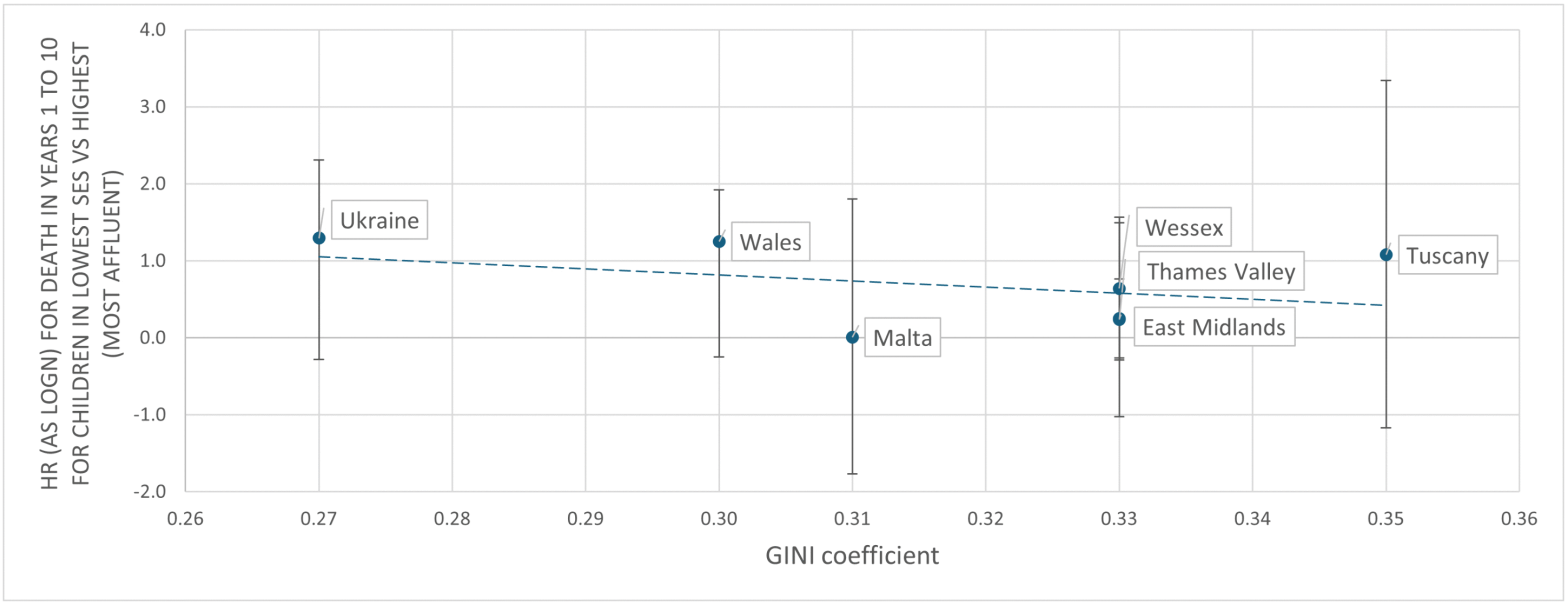
Socio-economic status differences in mortality ages 1-10 and GINI coefficient. Notes to Fig 5. CI 95% confidence interval, HR hazard ratio, logn natural logarithm, SES socio-economic status (GINI is named after its founder, Corrado Gini.). The natural logarithms of the hazard ratios and their error bars were plotted to allow visualisation of any relationship. The image is illustrative.

**Fig 6:**
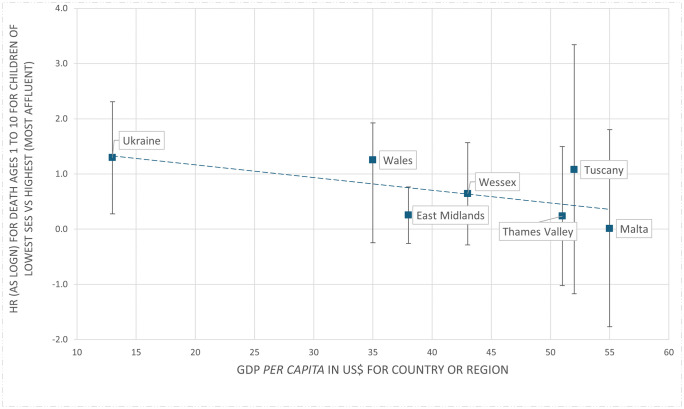
Socio-economic status differences in mortality ages 1-10 and GDP per capita. Notes to Fig 6. CI 95% confidence interval, GDP gross domestic product, HR hazard ratio, logn natural logarithm, SES socio-economic status. The natural logarithms of the hazard ratios and their error bars were plotted to allow visualisation of any relationship. The image is illustrative.

Scatter plots suggested that within-country differences in mortality according to SES are not ameliorated by lower income inequality, as assessed by the GINI coefficient for income (Fig 5), and may appear greater in countries with lower GDP *per capita* (Fig 6). The small numbers of deaths in some registers resulted in wide 95% confidence intervals. Trends were not statistically significant.

### Nationality and mortality

**Fig 7:**
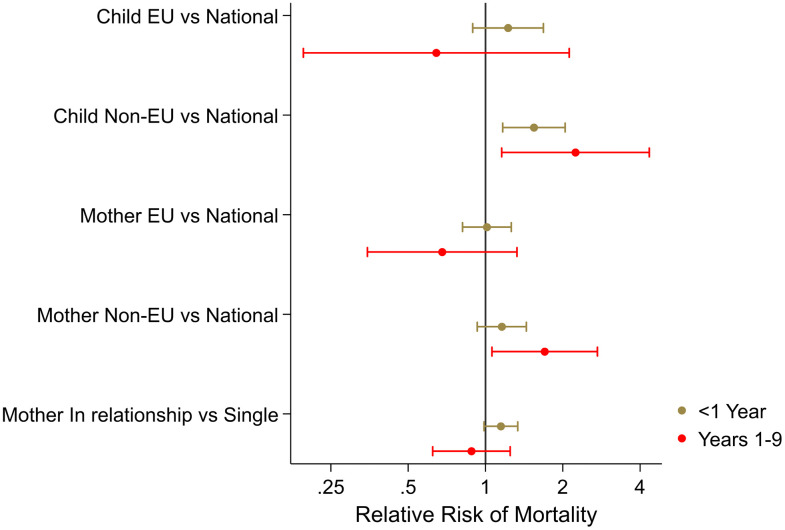
Mothers’ and Children’s nationality and mothers’ marital status in Finland and Emilia Romagna and relative risk of mortality.

Finland and Emilia Romagna contributed data on the nationality of 48,450 mothers and children. Survival was not affected if either the child or the mother was a national of an EU country other than the country of birth. Non-EU nationals were more vulnerable than EU nationals, particularly between ages 1 and 9 (Fig 7).

### Individual congenital anomalies

The associations between SES and CHD and severe CHD were stronger in childhood than infancy, and, in the UK, for the most deprived groups; this trend was less apparent in Southern Europe, particularly where there were few cases (S2 Table in S2 File). In infancy, in some countries, hazard ratios were high for *spina bifida*, hydrocephalus, congenital cataracts, gastroschisis, hydronephrosis, limb reduction, and craniosynostosis, but low numbers preclude interpretation. Deprivation was not associated with infant mortality for children with facial clefts, multicystic renal dysplasia or hypospadias (S3 Table in S3 File). The low numbers of deaths in children aged 1–10 precluded interpretable calculations. In some registers, associations between deprivation and mortality for children with CHD, severe microcephaly, limb defects, facial clefts or hydrocephalus were apparent (S4 Table in S3 File).

### Marital status and mortality

In the two registers with available data, Finland and Emilia Romagna, there was no evidence that children with single parents were more likely to die (Fig 7). Too few women were widowed for widowhood to be analysed.

## Discussion

Infants and children from the most deprived backgrounds were more likely to die than their contemporaries from affluent backgrounds, overall, as in the source populations (Table 1). The intermediate groups were less severely affected. SES and non-EU nationality appeared more closely associated with childhood (ages 1–10) than infant deaths. Hazard ratios were not uniform across Europe: associations between SES and mortality were consistent only in Ukraine and Wales. These were the countries with the lowest GDP *per capita*, but not the highest levels of income inequality, as assessed by GINI coefficients (Table 1). SES did not affect infant mortality in Funen, Denmark – the country with the highest GDP *per capita* and the lowest GINI coefficient*.*

### Ill and unequal

SES determines the survival of children with congenital anomalies in many, but not all, countries [9,12,15]. The greater effects of SES in older children, particularly in Wales and Ukraine, and for CHD, hydrocephalus, and severe microcephaly, contrast findings on CHD mortality [15]. The impact of maternal education decreased by age 10 in Tuscany, but not Ukraine. The increased mortality amongst non-EU nationals is consistent with earlier reports [59]. Early care of infants with anomalies from all backgrounds is based in specialist hospitals from birth. However, after the first year, there is less hospitalisation [60], and access to services is more dependent on primary care and the leverage of social capital [45,61]. While the physical environment in hospital is not influenced by SES, after discharge, material conditions (housing, heating, nutrition, cigarette smoke, alcohol use) are influenced by SES [62]. In Wales, but not England, the impact of SES of infant mortality differed little between infants with anomalies and the whole population. Data for childhood deaths indicate a sharper contrast between rich and poor amongst children with anomalies and the whole population (albeit sometimes based on different age ranges). Similarly, in Finland, the mortality hazard ratios for children aged 1–10 born outside the EU were higher than the rate ratios between high and low SES. Data are too sparse to speculate on the impact of resource constraints [63]. Respiratory conditions [64] and injury [65] are more common amongst children with anomalies, and are associated with mortality linked with housing standards and deprivation [66], placing the most deprived children with anomalies in double jeopardy.

In this study, English registers did not report statistically significant associations between mortality amongst children with major anomalies at ages 1–10 and area-based measures of deprivation. This is congruent with analyses considering infants with CHD post-operatively [67,68] but not analyses of CHD in all age groups [69]. These studies used data from all England [67–69], whereas the registers in this study covered only three regions of England, none of which are the most deprived [31].

### Total resources or unequal resources

Deaths in children aged under 5 contribute to inequalities in life expectancy [70]. In 2020, congenital anomalies accounted for 27.7% (uncertainty limits [UL] 26.6–28.8%) deaths in this age group in high income countries and 7.4% (UL 6.7–8.2%) elsewhere [71]. Infant and childhood mortality rates are no longer improving in high income countries [1,2,72]: they remained largely unchanged in Wales and England 2014–2023, at 0.39%, and increased between 2021 and 2022. The increase is attributed to more deaths in children from deprived backgrounds [28,73]; similar findings are reported in the USA [74].

Whether discrepancies in survival and life expectancy are driven predominantly by relative poverty and inequalities in income or resource constraints measured in absolute terms as GDP *per capita* is the subject of wider debate [75]. The association between SES and mortality amongst infants with CHD is more marked in the USA [76] than reported here for infants and children with the full range of major congenital anomalies, including CHD. This has been attributed to the USA’s high levels of income inequality (GINI coefficient 0.41 [35]) [16], but the structure of healthcare cannot be discounted. Our data are confined to Europe, but, within this cohort of the most vulnerable children, there was no suggestion that income equality was protective for infant and childhood mortality, congruent with international analyses [23–25]. This contextualisation of structural factors [77] reflects seminal work linking absolute, rather than relative, levels of poverty to health outcomes [78]: where there are insufficient resources, it becomes impossible to mitigate the effects of adverse environmental conditions [22]. The anomalies most affected by deprivation (CHD, severe CHD, Down syndrome (infants), limb defects, hydrocephalus, craniosynostosis, severe microcephaly, gastroschisis) were those requiring extensive surgery and rehabilitation. The anomalies least affected by deprivation, (hypospadias, multicystic renal dysplasia, Down syndrome (ages 1–10) often need less aftercare following initial surgery, reducing financial [63] and care demands [79].

Consequently, poor children are not only more likely to be born with congenital anomalies [62,80–82], they are more also more likely to die with them.

### Strengths and limitations

The EUROlinkCAT cohort is built on specialist registers with whole-population coverage. Consequently, it is free of volunteer bias and consequential collider bias [83]. All entries to EUROCAT registers are scrutinised and coded by experts using published criteria (EUROCAT guide 1.4, 1.5) [18] and linked to prospectively collected regional or national statistics. This avoids diagnostic imprecision and recall or social desirability response biases emanating from self-reporting, and any omissions of congenital anomalies from death certificates [84]. The diverse countries and regions contributing to this analysis cover almost the full spectrum of GDP *per capita* in Europe; however, most Eastern European countries did not participate. Many of the 23 EUROCAT registers were unable to link all infants to mortality records. Low numbers of deaths in some categories, including individual anomalies or nationality, and in some registers, restricted the data available for analysis.

The infant mortality rates reported by the EUROCAT registers (Table 3) were not closely aligned with the EUROSTAT data for mortality in all children, with or without anomalies (Table 1). Since >97% live-born children with major anomalies survive to age 1 and >96% to age 10 [20], where mortality rates in children with anomalies are substantially higher than infant mortality in the general population, it is possible that registers are under-reporting less severe anomalies. This analysis is confined to reported major anomalies. However, there is no evidence that reporting is influenced by SES. In Malta, termination of pregnancy for any reason, including lethal foetal anomalies, is illegal: this may explain the high infant mortality rates in this relatively affluent country. In Ukraine, the high population infant mortality may reflect causes of death not associated with anomalies [71]. We were unable to account for exposure to environmental pollution, which is associated with both higher prevalence of congenital anomalies [85,86] and lower SES in Wales [87] and Ukraine [88]. The persistent effects of caesium 137 (half-life 30 years) from the Chernobyl disaster, which affected the poorest regions and people of Ukraine, may account for some of the discrepancies in mortality rates reported here [88,89].

We were unable to report on population infant mortality according to SES for some registers. Data for childhood deaths were limited, with only Finland and Wales able to report deaths for ages 1–10.

SES and infant mortality are associated with many health, developmental and perinatal outcomes [90]. Associations between mortality and preterm birth, infant sex and maternal age are reported [91]. Information on some potential confounding factors was not available, including multiple births and maternal co-morbidities. Databases can only offer proxies for context. Lifestyle factors, such as exercise, sleep, smoking, obesity, alcohol use, recreational drug use, are not always recorded consistently. The use of SES as a proxy for these considerations warrants investigation within each database.

SES was recorded inconsistently across Europe: data from the European Deprivation Index [92] were not available. Differing measures of SES detract from international comparisons; some registers, such as Funen and Tuscany, using maternal education as a measure of SES, reported few deaths. Area-based deprivation scores do not fully account for discrepancies in income or education within an area; this is most marked in rural areas, where populations are dispersed. Individualised measures of SES (maternal education or occupation) mitigate the risk of the ecological fallacy [93] but omit the wider environmental context [94]. Both indices of multiple deprivation in this study include measures of the physical living environment, encompassing pollution, which affect health [95,96]. Future work should identify the impact of each component of the indices of multiple deprivation, including ‘access to services’ [97]. SES changes over time, which is difficult to capture in databases. Where records extended over 10 years, we do not know whether children moved to a different area or maternal education status changed.

Our analyses were confined to the most ill children in seven European countries: ~ 2.13% of European children have congenital anomalies [4], of whom only 3–4% die in childhood [20]. Sparse mortality data may represent the ‘tip of the iceberg’ of unmet needs attributable to deprivation, but should not be over-interpreted. There were too few events for exploration of many individual anomalies; these sparse data should be interpreted cautiously. Analyses are restricted to live births, excluding terminations of pregnancy, miscarriage or stillbirths. We do not know the extent to which these overall statistically significant findings and the international differences observed were due to differences in healthcare provision, healthcare resources, poverty, measures of SES or uncertainties surrounding the low numbers of events. Public domain data may facilitate data visualisation (Figs 5, 6), but inter-relationships based on population-level data are vulnerable to limitations of the indices [98,99], and the ecological fallacy [93]. However, linked data on mortality in children with diagnosed, verified major anomalies remain scarce, and these data offer leads for exploration. Analyses incorporating the full range of national wealth and income inequalities in Europe would inform long-standing debates [3,75,98].

### Implications

Deprivation was associated with higher mortality rates amongst children with congenital anomalies, overall. Differences were more pronounced amongst children aged 1–10 than infants, and greater in some countries than others. Our analyses indicate that, to achieve sustainable development goals, services should focus on the most vulnerable: children living with congenital anomalies and poverty.

## Supporting information

S1 File Record Statement. (DOCX)

S2 File S1 and S2 Tables. S1 Table. **Area Contrasts in Infant Mortality 2023 in English regions;** S2 Table. **Analysis of the effect of SES on survival up to 365 days and 365–3651 days for CHD and severe CHD.**
(DOCX)

S3 File S3 and S4 Tables. S3 Table. **Analysis of the effect of SES on survival up to 3654 days;** S4 Table. **Analysis of the effect of SES on survival 365–3651days.**
(XLSX)
