# Supplementary material for: Mortality amongst European children with congenital anomalies: Associations with socio-economic status in the EUROLINKCAT cohort
Source: PLoS One. 2026 Aug 5;21(8):e0352025. doi: 10.1371/journal.pone.0352025 (PMC13440827; doi:10.1371/journal.pone.0352025)
Supplement: Supplementary file 2 [file pone.0352025.s002.docx]

# S2 File. Supplementary tables

# Mortality amongst European Children with Congenital Anomalies: associations with Socio-economic Status in the EUROLINKCAT cohort

**S1 Table. Area Contrasts in Infant Mortality 2023 for English regions**

|  |  |  | Infant Mortality per 1,000 live births | Rate Ratio |  |
| --- | --- | --- | --- | --- | --- |
| Thames Valley | | W. Berkshire | 2.1 | 2.95 |  |
|  |  | Slough | 6.2 |  |  |
| EMSY | East Midlands | West Northamptonshire | 4.0 | 1.63 |  |
|  |  | Leicester | 6.5 |  |  |
|  | South Yorkshire | Rotherham | 1.8 | 3.17 |  |
|  |  | Sheffield | 5.7 |  |  |
| Wessex |  | Wiltshire | 2.3 | 3.04 |  |
|  |  | Havant | 7.0 |  |  |
| Highest and lowest infant mortality rates within geographical areas defined by ONS were extracted from ONS 2024. These may not be entirely contiguous with the borders on the regional registers of congenital anomalies. | | | | |  |
| ONS is unable to report infant mortality rates for all areas, due to sparsity of data. | | | | |  |
| Data are presented for illustration. | | | |  |  |

| **S2 Table. Analysis of the effect of SES on survival up to 364 days and days 365-3651 for CHD and severe CHD** | | | | | | | |
| --- | --- | --- | --- | --- | --- | --- | --- |
|  |  |  |  | **Infants** |  | **Ages 1-10** |  |
|  | **EUROCAT Subgroups** | | | **Congenital Heart Defects (al17)** | Severe CHD (al97) | **Congenital Heart Defects (al17)** | Severe CHD (al97) |
|  |  |  |  | HR (95% CI) | HR (95% CI) | HR (95% CI) | HR (95% CI) |
| Wales | Total in subgroup | | | 5798 | 1436 | 5728 | 1418 |
|  | Number non-missing | | | 5464 | 1335 | 5088 | 1162 |
|  | **least deprived** | Reference | | 1 | 1 | 1 | 1 |
|  | **most deprived group** | | | 1.31 (0.90-1.89) | 1.53 (0.91-2.6) | 3.08 (1.29-7.39) | 1.79 (0.65-4.93) |
|  | **intermediate group** | | | 1.15 (0.81-1.63) | 1.16 (0.70-1.90) | 1.63 (0.68-3.90) | 0.82 (0.30-2.25) |
|  | Wald test p-value (crude) | | | 0.34 | 0.17 | 0.01 | 0.09 |
| Thames Valley | Total in subgroup | | | 1154 | 419 | 1005 | 337 |
|  | Number non-missing | | | 1032 | 400 | 906 | 330 |
|  | **least deprived** | Reference | | 1 | 1 | 1 | 1 |
|  | **most deprived group** | | | 1.44 (0.66-3.16) | 1.85 (0.69-4.99) | 2.26 (0.44-11.58) | 3.79 (0.34-41.80) |
|  | **intermediate group** | | | 1.40 (0.90-2.18) | 1.51 (0.86-2.66) | 1.78 (0.63-5.06) | 3.83 (0.83-17.73) |
|  | Wald test p-value (crude) | | | 0.31 | 0.28 | 0.48 | 0.22 |
| Wessex | Total in subgroup | | | 1047 | 601 | 881 | 484 |
|  | Number non-missing | | | 965 | 560 | 828 | 461 |
|  | **least deprived** | Reference | | 1 | 1 | 1 | 1 |
|  | **most deprived group** | | | 1.99 (1.05-3.80) | 2.06 (0.94-4.52) | 1.63 (0.44-6.06) | 5.36 (0.48-59.75) |
|  | **intermediate group** | | | 1.45 (0.87-2.45) | 1.55 (0.83-2.90) | 0.45 (0.14-1.49) | 0.80 (0.07-8.80) |
|  | Wald test p-value (crude) | | | 0.11 | 0.19 | 0.12 | 0.13 |
| E Midlands, S Yorkshire | Total in subgroup | | | 2994 | 1303 | 2592 | 1053 |
|  | Number non-missing | | | 2776 | 1252 | 2449 | 1045 |
|  | **least deprived** | Reference | | 1 | 1 | 1 | 1 |
|  | **most deprived group** | | | 1.13 (0.76-1.70) | 1.07 (0.67-1.73) | 1.42 (0.65-3.13) | 2.08 (0.70-6.14) |
|  | **intermediate group** | | | 1.25 (0.85-1.83) | 1.09 (0.70-1.70) | 1.21 (0.56-2.60) | 1.46 (0.50-4.25) |
|  | Wald test p-value (crude) | | | 0.47 | 0.93 | 0.64 | 0.32 |
| Tuscany | Total in subgroup | | | 1737 | 350 | 1668 | 306 |
|  | Number non-missing | | | 1541 | 290 | 1492 | 262 |
|  | **least deprived** | Reference | | 1 | 1 | 1 | 1 |
|  | **most deprived group** | | | 3.03 (0.82-11.21) | 1.11 (0.14-9.03) | 8.77 (0.55-140.25) | 0.37 |
|  | **intermediate group** | | | 1.33 (0.64-2.79) | 1.20 (0.51-2.83) | 1.38 (0.15-12.37) | >1000 |
|  | Wald test p-value (crude) | | | 0.25 | 0.92 | 0.21 | 1.00 |
| Malta | Total in subgroup | | | 1294 | 253 | 1202 | 194 |
|  | Number non-missing | | | 381 | 87 | 349 | 67 |
|  | **least deprived** | Reference | | 1 | 1 | 1 | 1 |
|  | **most deprived group** | | | 0.71 (0.33-1.54) | 0.64 (0.26-1.62) | 0.58 (0.04-9.25) | 1.00 |
|  | **intermediate group** | | | 0.61 (0.23-1.62) | 0.20 (0.04-0.92) | 0.00 | 1.00 |
|  | Wald test p-value (crude) | | | 0.54 | 0.11 | 0.93 |  |
| Ukraine | Total in subgroup | | | 2026 | 511 | 1679 | 302 |
|  | Number non-missing | | | 2014 | 508 | 1670 | 302 |
|  | **least deprived** | Reference | | 1 | 1 | 1 | 1 |
|  | **most deprived group** | | | 1.65 (1.02-2.65) | 0.74 (0.35-1.58) | 2.33 (0.58-9.31) | 2.88 (0.48-17.23) |
|  | **intermediate group** | | | 1.78 (1.34-2.37) | 1.18 (0.83-1.67) | 1.87 (0.77-4.53) | 2.04 (0.60-6.91) |
|  | Wald test p-value (crude) | | | 0.00 | 0.32 | 0.33 | 0.43 |

HR adjusted hazard ratio, CHD congenital heart defects, CI confidence intervals

Only anomalies with >50 cases included.

Singletons and multiples included.

All multi-malformed groups included

Funen, Denmark were unable to provide data

Emilia Romagna data could not be incorporated into the meta-analysis.

Ages 1-10 taken as 365-3651 days
