# Supplementary material for: Mortality amongst European children with congenital anomalies: Associations with socio-economic status in the EUROLINKCAT cohort
Source: PLoS One. 2026 Aug 5;21(8):e0352025. doi: 10.1371/journal.pone.0352025 (PMC13440827; doi:10.1371/journal.pone.0352025)
Supplement: Supplementary file 3 [file pone.0352025.s005.docx]

# Response to reviewers

PONE-D-25-57012

Mortality amongst European Children with Congenital Anomalies: associations with Socio-economic Status in the EUROLINKCAT cohort

PLOS One

Dear Dr. Jordan,

Thank you for submitting your manuscript to PLOS ONE. After careful consideration, we feel that it has merit but does not fully meet PLOS ONE’s publication criteria as it currently stands. Therefore, we invite you to submit a revised version of the manuscript that addresses the points raised during the review process.

Please submit your revised manuscript by May 15 2026 11:59PM. If you will need more time than this to complete your revisions, please reply to this message or contact the journal office at [plosone@plos.org](mailto:plosone@plos.org). When you're ready to submit your revision, log on to <https://www.editorialmanager.com/pone/> and select the 'Submissions Needing Revision' folder to locate your manuscript file.

Please include the following items when submitting your revised manuscript:

- A letter that responds to each point raised by the academic editor and reviewer(s). You should upload this letter as a separate file labeled 'Response to Reviewers'.
- A marked-up copy of your manuscript that highlights changes made to the original version. You should upload this as a separate file labeled 'Revised Manuscript with Track Changes'.
- An unmarked version of your revised paper without tracked changes. You should upload this as a separate file labeled 'Manuscript'.

If you would like to make changes to your financial disclosure, please include your updated statement in your cover letter. Guidelines for resubmitting your figure files are available below the reviewer comments at the end of this letter.

If applicable, we recommend that you deposit your laboratory protocols in protocols.io to enhance the reproducibility of your results. Protocols.io assigns your protocol its own identifier (DOI) so that it can be cited independently in the future. For instructions see: [https://journals.plos.org/plosone/s/submission-guidelines#loc-laboratory-protocols](https://eur03.safelinks.protection.outlook.com/?url=https%3A%2F%2Ftrack.editorialmanager.com%2FCL0%2Fhttps%3A%252F%252Fjournals.plos.org%252Fplosone%252Fs%252Fsubmission-guidelines%2523loc-laboratory-protocols%2F1%2F010f019d42494cb4-88ff2a99-21f7-4470-89cb-2b164e87d891-000000%2FHY7dIkOMSCtfaD_pNsWGeXAFGgMZhkBdabJsqh1ioH4%3D256&data=05%7C02%7Cs.e.jordan%40swansea.ac.uk%7Cf0c24c27f4d248b0194d08de8ee347ce%7Cbbcab52e9fbe43d6a2f39f66c43df268%7C0%7C0%7C639105304274546496%7CUnknown%7CTWFpbGZsb3d8eyJFbXB0eU1hcGkiOnRydWUsIlYiOiIwLjAuMDAwMCIsIlAiOiJXaW4zMiIsIkFOIjoiTWFpbCIsIldUIjoyfQ%3D%3D%7C0%7C%7C%7C&sdata=GsvD99jsJEjb9XMaZ5fw6MZCADsD1xOoZLdQLxPmkS4%3D&reserved=0). Additionally, PLOS ONE offers an option for publishing peer-reviewed Lab Protocol articles, which describe protocols hosted on protocols.io. Read more information on sharing protocols at [https://plos.org/protocols?utm_medium=editorial-email&utm_source=authorletters&utm_campaign=protocols](https://eur03.safelinks.protection.outlook.com/?url=https%3A%2F%2Ftrack.editorialmanager.com%2FCL0%2Fhttps%3A%252F%252Fplos.org%252Fprotocols%253Futm_medium%3Deditorial-email%2526utm_source%3Dauthorletters%2526utm_campaign%3Dprotocols%2F1%2F010f019d42494cb4-88ff2a99-21f7-4470-89cb-2b164e87d891-000000%2F47wAjfCtRqWQSV6KmCw0zBbXj4hFx_NUBxGmBlv04z0%3D256&data=05%7C02%7Cs.e.jordan%40swansea.ac.uk%7Cf0c24c27f4d248b0194d08de8ee347ce%7Cbbcab52e9fbe43d6a2f39f66c43df268%7C0%7C0%7C639105304274564705%7CUnknown%7CTWFpbGZsb3d8eyJFbXB0eU1hcGkiOnRydWUsIlYiOiIwLjAuMDAwMCIsIlAiOiJXaW4zMiIsIkFOIjoiTWFpbCIsIldUIjoyfQ%3D%3D%7C0%7C%7C%7C&sdata=qKXZnddcN9AMWn%2Fel3%2Bk%2B7yDbJCCsF%2F%2FK72nqGtG%2BGo%3D&reserved=0).

As the corresponding author, your ORCID iD is verified in the submission system and will appear in the published article. PLOS supports the use of ORCID, and we encourage all coauthors to register for an ORCID iD and use it as well. Please encourage your coauthors to verify their ORCID iD within the submission system before final acceptance, as unverified ORCID iDs will not appear in the published article. *Only* the individual author can complete the verification step; PLOS staff *cannot* verify ORCID iDs on behalf of authors.

We look forward to receiving your revised manuscript.

Kind regards,

Hakan Aylanc
Academic Editor
PLOS One

Additional Editor Comments :

We look forward to receiving your revisions as soon as possible, in response to the reviewers' comments. Best of wishes.

Dear Professor Aylanc,

Thank you for these constructive comments. We have added further data, as the reviewer suggests: this strengthen the manuscript.

Our responses are below.

Journal Requirements:

When submitting your revision, we need you to address these additional requirements.
1. Please ensure that your manuscript meets PLOS ONE's style requirements, including those for file naming. The PLOS ONE style templates can be found at
[https://journals.plos.org/plosone/s/file?id=wjVg/PLOSOne_formatting_sample_main_body.pdf](https://eur03.safelinks.protection.outlook.com/?url=https%3A%2F%2Ftrack.editorialmanager.com%2FCL0%2Fhttps%3A%252F%252Fjournals.plos.org%252Fplosone%252Fs%252Ffile%253Fid%3DwjVg%252FPLOSOne_formatting_sample_main_body.pdf%2F1%2F010f019d42494cb4-88ff2a99-21f7-4470-89cb-2b164e87d891-000000%2FnNlUvuXGzO81WAeXGCijHkwLf8IXy0zpQE6YKpRmfIs%3D256&data=05%7C02%7Cs.e.jordan%40swansea.ac.uk%7Cf0c24c27f4d248b0194d08de8ee347ce%7Cbbcab52e9fbe43d6a2f39f66c43df268%7C0%7C0%7C639105304274578831%7CUnknown%7CTWFpbGZsb3d8eyJFbXB0eU1hcGkiOnRydWUsIlYiOiIwLjAuMDAwMCIsIlAiOiJXaW4zMiIsIkFOIjoiTWFpbCIsIldUIjoyfQ%3D%3D%7C0%7C%7C%7C&sdata=%2FksCztBlWhf1eD7%2BaXNPi%2F7RGDamjhZRckMpRzkRZQQ%3D&reserved=0) and [https://journals.plos.org/plosone/s/file?id=ba62/PLOSOne_formatting_sample_title_authors_affiliations.pdf](https://eur03.safelinks.protection.outlook.com/?url=https%3A%2F%2Ftrack.editorialmanager.com%2FCL0%2Fhttps%3A%252F%252Fjournals.plos.org%252Fplosone%252Fs%252Ffile%253Fid%3Dba62%252FPLOSOne_formatting_sample_title_authors_affiliations.pdf%2F1%2F010f019d42494cb4-88ff2a99-21f7-4470-89cb-2b164e87d891-000000%2Fpk89le6cfGJv60SMeIBeYdnAD7QEOaFambMnFamr2Js%3D256&data=05%7C02%7Cs.e.jordan%40swansea.ac.uk%7Cf0c24c27f4d248b0194d08de8ee347ce%7Cbbcab52e9fbe43d6a2f39f66c43df268%7C0%7C0%7C639105304274596050%7CUnknown%7CTWFpbGZsb3d8eyJFbXB0eU1hcGkiOnRydWUsIlYiOiIwLjAuMDAwMCIsIlAiOiJXaW4zMiIsIkFOIjoiTWFpbCIsIldUIjoyfQ%3D%3D%7C0%7C%7C%7C&sdata=%2Bd1QOTcFc2KnrnVicOknL%2BFfjpkG6XISPCjXP8AC6RI%3D&reserved=0)

**We have endeavoured to comply. Please let us know of any further changes needed.**

2. Your ethics statement should only appear in the Methods section of your manuscript. If your ethics statement is written in any section besides the Methods, please delete it from any other section.

***We apologise, the extraneous information has been removed.***

3. We note that you have indicated that there are restrictions to data sharing for this study. PLOS only allows data to be available upon request if there are legal or ethical restrictions on sharing data publicly. For more information on unacceptable data access restrictions, please see <http://journals.plos.org/plosone/s/data-availability#loc-unacceptable-data-access-restrictions>.

Data availability

Before we proceed with your manuscript, please address the following prompts:

a) If there are ethical or legal restrictions on sharing a de-identified data set, please explain them in detail (e.g., data contain potentially identifying or sensitive patient information, data are owned by a third-party organization, etc.) and who has imposed them (e.g., a Research Ethics Committee or Institutional Review Board, etc.). Please also provide contact information for a data access committee, ethics committee, or other institutional body to which data requests may be sent.

***We have revised our data availability statement. This is now aligned with statements from other papers from the Eurolinkcat study published in PLOS One (Loane et al 2023, 2021):***

## Data availability statement

***All data related to these analyses are contained within the paper and supplementary information.***

***We are legally not allowed to share the third-party administrative data used in this study as it belongs to the data providers in each of the regions i.e. the regional or national statistical organisations. The study team had access to aggregate data only from each region i.e. the linked patient level data remained in the local region. The authors did not receive any special privileges in accessing the data, and were obliged to abide by the same rules and regulations pertaining to the data as other researchers. All the variables included in the study are found in the pdf for WP3 (Loane et al 2019). This includes the variable names, description, format, and coding scheme. All our documentation is available on the EUROlinkCAT website (***[***http://www.EUROlinkCAT.eu/wp2-buildingresultsrepository***](http://www.eurolinkcat.eu/wp2-buildingresultsrepository)***). We encourage any interested parties to apply to the EUROlinkCAT management team to assist them in obtaining approval from the data providers in each region/ country to use the aggregated data for an approved study***  [***http://www.EUROlinkCAT.eu/contactinformationanddatarequests***](http://www.eurolinkcat.eu/contactinformationanddatarequests)***.***

***Eurolinkcat data may be requested from Dr. Maria Loane, via a data request form available:***

[EUROlinkCAT - Establishing a linked European Cohort of Children with Congenital Anomalies](https://www.eurolinkcat.eu/contactinformationanddatarequests)

***The data that support the findings of this study are available from the participating registries of congenital anomalies, but restrictions apply to the availability of these data, which were used under license for the current study, and so are not publicly available. Data are however available from the authors for scientifically valid requests and with permission of the participating registries of congenital anomalies.***

b) If there are no restrictions, please upload the minimal anonymized data set necessary to replicate your study findings to a stable, public repository and provide us with the relevant URLs, DOIs, or accession numbers. For a list of recommended repositories, please see
<https://journals.plos.org/plosone/s/recommended-repositories>. You also have the option of uploading the data as Supporting Information files, but we would recommend depositing data directly to a data repository if possible.

We will update your Data Availability statement on your behalf to reflect the information you provide.

4. In the online submission form, you indicated that data were used under license for the current study. Because restrictions apply to the availability of these data, there are no publicly available data. Data can be released from the authors for scientifically valid requests, upon permission of the participating registers of congenital anomalies.

All PLOS journals now require all data underlying the findings described in their manuscript to be freely available to other researchers, either 1. In a public repository, 2. Within the manuscript itself, or 3. Uploaded as supplementary information.
This policy applies to all data except where public deposition would breach compliance with the protocol approved by your research ethics board. If your data cannot be made publicly available for ethical or legal reasons (e.g., public availability would compromise patient privacy), please explain your reasons on resubmission and your exemption request will be escalated for approval.

***There are legal, governance and ethical constraints on the release of EUROLINKCAT data to avoid disclosure of identifiable information about small numbers of vulnerable families.***

## Data availability statement

***This has been revised, as above***

b) If there are no restrictions, please upload the minimal anonymized data set necessary to replicate your study findings to a stable, public repository and provide us with the relevant URLs, DOIs, or accession numbers. For a list of recommended repositories, please see
<https://journals.plos.org/plosone/s/recommended-repositories>. You also have the option of uploading the data as Supporting Information files, but we would recommend depositing data directly to a data repository if possible.

We will update your Data Availability statement on your behalf to reflect the information you provide.

4. In the online submission form, you indicated that data were used under license for the current study. Because restrictions apply to the availability of these data, there are no publicly available data. Data can be released from the authors for scientifically valid requests, upon permission of the participating registers of congenital anomalies.

All PLOS journals now require all data underlying the findings described in their manuscript to be freely available to other researchers, either 1. In a public repository, 2. Within the manuscript itself, or 3. Uploaded as supplementary information.
This policy applies to all data except where public deposition would breach compliance with the protocol approved by your research ethics board. If your data cannot be made publicly available for ethical or legal reasons (e.g., public availability would compromise patient privacy), please explain your reasons on resubmission and your exemption request will be escalated for approval.

***We have revised our data availability statement. This is now aligned with statements from other papers from the Eurolinkcat study published in PLOS One (Loane et al 2023, 2021):***

## Please see ‘Data availability statement’ above

 5. Please amend your authorship list in your manuscript file to include author Lyuba Ostapchuk, Wladimir Wertrlecki.
 ***We apologize for any typographical error. Both these authors are listed on the first page of the manuscript file. I have sent a query to the journal’s office.***

6. Please amend the manuscript submission data (via Edit Submission) to include author Lyubov Ostapchuk, Wertelecki W.

***We apologise for any omission. I have checked on the submission files, and these authors are listed. However, their institution (***Rivne Medical Diagnostic Center and OMNI-Net Ukraine) ***is unverified. I am unable to change the institution. Please could you advise?***

7. Please amend your list of authors on the manuscript to ensure that each author is linked to an affiliation. Authors’ affiliations should reflect the institution where the work was done (if authors moved subsequently, you can also list the new affiliation stating “current affiliation:….” as necessary).

***We apologise for any omission. We have adjusted Daniel Thayer’s location.***

 8. If the reviewer comments include a recommendation to cite specific previously published works, please review and evaluate these publications to determine whether they are relevant and should be cited. There is no requirement to cite these works unless the editor has indicated otherwise.

NA

Additional Editor Comments :

We look forward to receiving your revisions as soon as possible, in response to the reviewers' comments. Best of wishes.

[Note: HTML markup is below. Please do not edit.]

Reviewers' comments:

Reviewer's Responses to Questions

**Comments to the Author**

1. Is the manuscript technically sound, and do the data support the conclusions?

The manuscript must describe a technically sound piece of scientific research with data that supports the conclusions. Experiments must have been conducted rigorously, with appropriate controls, replication, and sample sizes. The conclusions must be drawn appropriately based on the data presented.

Reviewer #1: Partly

2. Has the statistical analysis been performed appropriately and rigorously?

Reviewer #1: Yes

3. Have the authors made all data underlying the findings in their manuscript fully available?

The [PLOS Data policy](https://eur03.safelinks.protection.outlook.com/?url=http%3A%2F%2Ftrack.editorialmanager.com%2FCL0%2Fhttp%3A%252F%252Fwww.plosone.org%252Fstatic%252Fpolicies.action%2523sharing%2F1%2F010f019d42494cb4-88ff2a99-21f7-4470-89cb-2b164e87d891-000000%2FgAQj503iqgrQV_eUbwZsqFEkxvuHh1bNhi_D4sF0zVs%3D256&data=05%7C02%7Cs.e.jordan%40swansea.ac.uk%7Cf0c24c27f4d248b0194d08de8ee347ce%7Cbbcab52e9fbe43d6a2f39f66c43df268%7C0%7C0%7C639105304274788230%7CUnknown%7CTWFpbGZsb3d8eyJFbXB0eU1hcGkiOnRydWUsIlYiOiIwLjAuMDAwMCIsIlAiOiJXaW4zMiIsIkFOIjoiTWFpbCIsIldUIjoyfQ%3D%3D%7C0%7C%7C%7C&sdata=ns0URl3uVVoVzIFRrrGX3SOJIsauxUEvtp1GWlucBbg%3D&reserved=0) requires authors to make all data underlying the findings described in their manuscript fully available without restriction, with rare exception (please refer to the Data Availability Statement in the manuscript PDF file). The data should be provided as part of the manuscript or its supporting information, or deposited to a public repository. For example, in addition to summary statistics, the data points behind means, medians and variance measures should be available. If there are restrictions on publicly sharing data—e.g. participant privacy or use of data from a third party—those must be specified.

Reviewer #1: No

4. Is the manuscript presented in an intelligible fashion and written in standard English?

PLOS ONE does not copyedit accepted manuscripts, so the language in submitted articles must be clear, correct, and unambiguous. Any typographical or grammatical errors should be corrected at revision, so please note any specific errors here.

Reviewer #1: Yes

5. Review Comments to the Author

Please use the space provided to explain your answers to the questions above. You may also include additional comments for the author, including concerns about dual publication, research ethics, or publication ethics. (Please upload your review as an attachment if it exceeds 20,000 characters)

Reviewer #1: This manuscript investigates the impact of socio-economic status (SES) on mortality among children with major congenital anomalies up to age 10, using data from the EUROlinkCAT cohort. The study addresses a significant gap in the literature by extending the analysis beyond infancy. The use of a large, multi country cohort is a major strength.

***Thank you for your support.***

However, there are significant methodological concerns regarding the heterogeneity of the study population and the lack of a reference group that need to be addressed to validly interpret the findings.

| Major Revisions: 1-Stratification by Anomaly Subgroups: The current analysis treats major congenital anomalies as a single, homogenous group; however, from a clinical and genetic perspective, this approach obscures critical differences. The natural history, mortality risk, and dependency on high resource medical interventions vary drastically across diverse categories, ranging from complex congenital heart defects and neural tube defects to chromosomal abnormalities and isolated limb defects.  Therefore, the authors should perform a subgroup analysis stratified by major organ systems or broad anomaly categories, such as isolated congenital heart defects, chromosomal anomalies, and non-chromosomal structural defects. Even if sample sizes limit granular analysis, this broad stratification is essential to determine if the SES gradient is driven by specific, resource dependent pathologies. | We agree, and thank you for this suggestion.  This study is restricted to major congenital anomalies, as defined by EUROCAT: major congenital anomalies are structural changes that have significant medical, surgical, social or cosmetic consequences for the affected individual, and typically require medical intervention. Nevertheless, variation exists within and between diagnostic categories, sometimes depending on the severity of the lesion (for *spina bifida* this is the level of the lesion). Unfortunately, EUROCAT registers are unable to capture severity, and hence resource demands.    With this *caveat, w*e now tabulate the full range of anomalies, where there are adequate numbers to work within governance approvals, in a supplementary file S3 file. S3, S4 Tables. However, the low number of deaths precluded analysis for many anomalies, as elsewhere (Tan et al 2023). In most countries, CHD and severe CHD had sufficient subjects for analysis, and the results are tabulated separately in S2 file, S2 Table.  We have written under methods, line 187:  Cox proportional hazard ratios in individual congenital anomalies were reported for individual countries where >50 deaths occurred, without meta-analysis (Austin et al 2017).  Under results, line 284 *et seq.*: The associations between SES and CHD and severe CHD were stronger in childhood than infancy, and, in the UK, for the most deprived groups; this trend was less apparent in Southern Europe, particularly where there were few cases (S2 Table). In infancy, in some countries, hazard ratios were high for *spina bifida*, hydrocephalus, congenital cataracts, gastroschisis, hydronephrosis, limb reduction, and craniosynostosis, but low numbers preclude interpretation. Deprivation was not associated with infant mortality for children with facial clefts, multicystic renal dysplasia or hypospadias (S3 Table). The low numbers of deaths in children aged 1-10 precluded interpretable calculations. In some registers, associations between CHD, severe microcephaly, limb defects, facial clefts or hydrocephalus and childhood mortality were apparent (S4 Table).  Under discussion, line 352 *et seq. :*  The anomalies most affected by deprivation (CHD, severe CHD, Down syndrome (infants), limb defects, hydrocephalus, craniosynostosis, severe microcephaly, gastroschisis) were those requiring extensive surgery and rehabilitation. The anomalies least affected by deprivation, (hypospadias, multicystic renal dysplasia, Down syndrome (ages 1-10) often need less aftercare following initial surgery, reducing financial [63] and care demands [79]. |
| --- | --- |
|  |  |
| 2-The study analyzes mortality differences within the congenital anomaly cohort but lacks a comparison with the background mortality of children without anomalies. This reference group is necessary to distinguish whether low SES exerts a disproportionately higher negative effect on children with anomalies compared to their healthy peers or merely reflects known background inequalities. The authors should include mortality rates for children without congenital anomalies from the same regions to demonstrate if the socioeconomic mortality gap is significantly wider in the anomaly group. | Thank you for this suggestion. We agree this is important. We sought population data from the public domain, and the register leads to report infant and childhood mortality according to SES. Where available, this has been added to Table 1. We note that EUROSTAT do not report these data. This accentuates the importance of our data. The sparsity of data limits interpretation, and we acknowledge this. International inconsistency in reporting of SES is an acknowledged limitation.  We have explained under ‘settings’, acknowledging the contribution of congenital anomalies to deaths in infancy and childhood, line 108:  Population infant mortality, and SES gradations in infant and childhood mortality rates were reported to illustrate comparability with whole-population mortality (Table 1); some 20-31% infant and childhood deaths are attributed to congenital anomalies [PHW 2022, Statistics Finland nd, ONS 2025].    We have commented under ‘discussion’, line 319 *et seq.*:  In Wales, but not England, the impact of SES of infant mortality differed little between infants with anomalies and the whole population. Data for childhood deaths indicate a sharper contrast between rich and poor amongst children with anomalies and the whole population (albeit sometimes based on different age ranges). Similarly, in Finland, the mortality hazard ratios for children aged 1-9 born outside the EU were higher than the rate ratios between high and low SES. Data are too sparse to speculate on the impact of resource constraints [63]  We acknowledge, line 385:  We were unable to report on population infant mortality according to SES for some registers. Data for childhood deaths were limited, with only Finland and Wales able to report deaths for ages 1-9. |

## References

Austin PC, Allignol A, Fine JP. The number of primary events per variable affects estimation of the subdistribution hazard competing risks model. Journal of Clinical Epidemiology. 2017;83: 75–84. doi:10.1016/j.jclinepi.2016.11.017

Kim SW, Siljehav V, Seppänen AV, Maier RF, Varendi H, Draper E, Andronis L, Cuttini M, Zeitlin J, Petrou S; SHIPS Research Group. Economic costs at age five for very preterm children with congenital anomalies: evidence from a European cohort. Pediatr Res. 2026 Jan 7. doi: 10.1038/s41390-025-04672-z. Epub ahead of print. PMID: 41501409. (Table 2 has data)

Loane M, Densem J, de Walle H, Garne E, Given J, Karnell K, Pierini A, Rankin J, Reid A, Rissmann A, Tan J, Morris JK (2019). EUROlinkCAT Common Data Model: Survival. Ulster University. DOI: 10.13140/RG.2.2.21187.84005

Loane M, Densem J, de Walle H, Garne E, Given J, Karnell K, Pierini A, Rankin J, Reid A, Rissmann A, Tan J, Morris JK (2019). EUROlinkCAT Common Data Model: Survival. Ulster University. DOI: 10.13140/RG.2.2.21187.84005 [https://www.eurolinkcat.eu/wp2-buildingresultsrepository/eurolinkcatpubliccommondatamodels](https://eur03.safelinks.protection.outlook.com/?url=https%3A%2F%2Fwww.eurolinkcat.eu%2Fwp2-buildingresultsrepository%2Feurolinkcatpubliccommondatamodels&data=05%7C02%7CS.E.Jordan%40Swansea.ac.uk%7Ceabb9c9b455543545a8a08dea7ac3949%7Cbbcab52e9fbe43d6a2f39f66c43df268%7C0%7C0%7C639132555466882532%7CUnknown%7CTWFpbGZsb3d8eyJFbXB0eU1hcGkiOnRydWUsIlYiOiIwLjAuMDAwMCIsIlAiOiJXaW4zMiIsIkFOIjoiTWFpbCIsIldUIjoyfQ%3D%3D%7C0%7C%7C%7C&sdata=zN6k7sL%2Bei6v4lD4rOWzcIme4ox0bsvyXd3jtL6A304%3D&reserved=0)

Loane M, Given JE, Tan J, Barišić I, Barrachina-Bonet L, Cavero-Carbonell C, Coi A, Densem J, Garne E, Gissler M, Heino A, Jordan S, Lutke R, Neville AJ, Odak L, Puccini A, Santoro M, Scanlon I, Urhoj SK, de Walle HEK, Wellesley D, Morris JK. Creating a population-based cohort of children born with and without congenital anomalies using birth data matched to hospital discharge databases in 11 European regions: Assessment of linkage success and data quality. PLoS One. 2023 Aug 30;18(8):e0290711. doi: 10.1371/journal.pone.0290711. PMID: 37647348; PMCID: PMC10468043.

Loane M, Given JE, Tan J, Reid A, Akhmedzhanova D, Astolfi G, Barišić I, Bertille N, Bonet LB, Carbonell CC, Carollo OM, Coi A, Densem J, Draper E, Garne E, Gatt M, Glinianaia SV, Heino A, Hond ED, Jordan S, Khoshnood B, Kiuru-Kuhlefelt S, Klungsøyr K, Lelong N, Lutke LR, Neville AJ, Ostapchuk L, Puccini A, Rissmann A, Santoro M, Scanlon I, Thys G, Tucker D, Urhoj SK, de Walle HEK, Wellesley D, Zurriaga O, Morris JK. Linking a European cohort of children born with congenital anomalies to vital statistics and mortality records: A EUROlinkCAT study. PLoS One. 2021 Aug 27;16(8):e0256535. doi: 10.1371/journal.pone.0256535. PMID: 34449798; PMCID: PMC8396745.

ONS 2025 **Child and infant mortality in England and Wales: 2023** [**Child and infant mortality in England and Wales - Office for National Statistics**](https://www.ons.gov.uk/peoplepopulationandcommunity/birthsdeathsandmarriages/deaths/bulletins/childhoodinfantandperinatalmortalityinenglandandwales/2023)

PHW 2022 Patterns and trends of child deaths in Wales, 2011-2020 [phw.nhs.wales/publications/publications1/patterns-and-trends-of-child-deaths-in-wales-2011-2020/](https://phw.nhs.wales/publications/publications1/patterns-and-trends-of-child-deaths-in-wales-2011-2020/)

**Statistics Finland** <https://pxdata.stat.fi/PxWeb/pxweb/en/StatFin/StatFin__ksyyt/statfin_ksyyt_pxt_11bd.px/>

 Tan J, Glinianaia SV, Rankin J, Pierini A, Santoro M, Coi A, et al. Risk factors for mortality in infancy and childhood in children with major congenital anomalies: A European population-based cohort study. Paediatric and Perinatal Epidemiology. 2023;37: 679–690. doi:10.1111/ppe.13010

Vaitėnienė EM, Jakaitienė A, Murauskienė L, Utkus A. Cost analysis of financial, productivity, and informal care burdens in families raising children with congenital anomalies. Health Econ Rev. 2026 Jan 24;16(1):19. doi: 10.1186/s13561-026-00729-9. PMID: 41579276; PMCID: PMC12910806.

To ensure your figures meet our technical requirements, please review our figure guidelines: <https://journals.plos.org/plosone/s/figures>

You may also use PLOS’s free figure tool, NAAS, to help you prepare publication quality figures: <https://journals.plos.org/plosone/s/figures#loc-tools-for-figure-preparation>.

NAAS will assess whether your figures meet our technical requirements by comparing each figure against our figure specifications.

From email of 31.3.26

When submitting your revision, we need you to address these additional requirements.
1. Please ensure that your manuscript meets PLOS ONE's style requirements, including those for file naming. The PLOS ONE style templates can be found at
[https://journals.plos.org/plosone/s/file?id=wjVg/PLOSOne_formatting_sample_main_body.pdf](https://eur03.safelinks.protection.outlook.com/?url=https%3A%2F%2Ftrack.editorialmanager.com%2FCL0%2Fhttps%3A%252F%252Fjournals.plos.org%252Fplosone%252Fs%252Ffile%253Fid%3DwjVg%252FPLOSOne_formatting_sample_main_body.pdf%2F1%2F010f019d42494cb4-88ff2a99-21f7-4470-89cb-2b164e87d891-000000%2FnNlUvuXGzO81WAeXGCijHkwLf8IXy0zpQE6YKpRmfIs%3D256&data=05%7C02%7Cs.e.jordan%40swansea.ac.uk%7Cf0c24c27f4d248b0194d08de8ee347ce%7Cbbcab52e9fbe43d6a2f39f66c43df268%7C0%7C0%7C639105304274578831%7CUnknown%7CTWFpbGZsb3d8eyJFbXB0eU1hcGkiOnRydWUsIlYiOiIwLjAuMDAwMCIsIlAiOiJXaW4zMiIsIkFOIjoiTWFpbCIsIldUIjoyfQ%3D%3D%7C0%7C%7C%7C&sdata=%2FksCztBlWhf1eD7%2BaXNPi%2F7RGDamjhZRckMpRzkRZQQ%3D&reserved=0) and [https://journals.plos.org/plosone/s/file?id=ba62/PLOSOne_formatting_sample_title_authors_affiliations.pdf](https://eur03.safelinks.protection.outlook.com/?url=https%3A%2F%2Ftrack.editorialmanager.com%2FCL0%2Fhttps%3A%252F%252Fjournals.plos.org%252Fplosone%252Fs%252Ffile%253Fid%3Dba62%252FPLOSOne_formatting_sample_title_authors_affiliations.pdf%2F1%2F010f019d42494cb4-88ff2a99-21f7-4470-89cb-2b164e87d891-000000%2Fpk89le6cfGJv60SMeIBeYdnAD7QEOaFambMnFamr2Js%3D256&data=05%7C02%7Cs.e.jordan%40swansea.ac.uk%7Cf0c24c27f4d248b0194d08de8ee347ce%7Cbbcab52e9fbe43d6a2f39f66c43df268%7C0%7C0%7C639105304274596050%7CUnknown%7CTWFpbGZsb3d8eyJFbXB0eU1hcGkiOnRydWUsIlYiOiIwLjAuMDAwMCIsIlAiOiJXaW4zMiIsIkFOIjoiTWFpbCIsIldUIjoyfQ%3D%3D%7C0%7C%7C%7C&sdata=%2Bd1QOTcFc2KnrnVicOknL%2BFfjpkG6XISPCjXP8AC6RI%3D&reserved=0)

2. Your ethics statement should only appear in the Methods section of your manuscript. If your ethics statement is written in any section besides the Methods, please delete it from any other section.

3. We note that you have indicated that there are restrictions to data sharing for this study. PLOS only allows data to be available upon request if there are legal or ethical restrictions on sharing data publicly. For more information on unacceptable data access restrictions, please see <http://journals.plos.org/plosone/s/data-availability#loc-unacceptable-data-access-restrictions>.

Before we proceed with your manuscript, please address the following prompts:

a) If there are ethical or legal restrictions on sharing a de-identified data set, please explain them in detail (e.g., data contain potentially identifying or sensitive patient information, data are owned by a third-party organization, etc.) and who has imposed them (e.g., a Research Ethics Committee or Institutional Review Board, etc.). Please also provide contact information for a data access committee, ethics committee, or other institutional body to which data requests may be sent.

b) If there are no restrictions, please upload the minimal anonymized data set necessary to replicate your study findings to a stable, public repository and provide us with the relevant URLs, DOIs, or accession numbers. For a list of recommended repositories, please see
<https://journals.plos.org/plosone/s/recommended-repositories>. You also have the option of uploading the data as Supporting Information files, but we would recommend depositing data directly to a data repository if possible.

We will update your Data Availability statement on your behalf to reflect the information you provide.

4. In the online submission form, you indicated that data were used under license for the current study. Because restrictions apply to the availability of these data, there are no publicly available data. Data can be released from the authors for scientifically valid requests, upon permission of the participating registers of congenital anomalies.

All PLOS journals now require all data underlying the findings described in their manuscript to be freely available to other researchers, either 1. In a public repository, 2. Within the manuscript itself, or 3. Uploaded as supplementary information.
This policy applies to all data except where public deposition would breach compliance with the protocol approved by your research ethics board. If your data cannot be made publicly available for ethical or legal reasons (e.g., public availability would compromise patient privacy), please explain your reasons on resubmission and your exemption request will be escalated for approval.

5. Please amend your authorship list in your manuscript file to include author Lyuba Ostapchuk, Wladimir Wertrlecki.

6. Please amend the manuscript submission data (via Edit Submission) to include author Lyubov Ostapchuk, Wertelecki W.

7. Please amend your list of authors on the manuscript to ensure that each author is linked to an affiliation. Authors’ affiliations should reflect the institution where the work was done (if authors moved subsequently, you can also list the new affiliation stating “current affiliation:….” as necessary).

8. If the reviewer comments include a recommendation to cite specific previously published works, please review and evaluate these publications to determine whether they are relevant and should be cited. There is no requirement to cite these works unless the editor has indicated otherwise.
