# Supplementary material for: Mortality amongst European children with congenital anomalies: Associations with socio-economic status in the EUROLINKCAT cohort
Source: PLoS One. 2026 Aug 5;21(8):e0352025. doi: 10.1371/journal.pone.0352025 (PMC13440827; doi:10.1371/journal.pone.0352025)
Supplement: Peer review history

# Peer review: Decision Letter 0

**DOI:** 10.1371/journal.pone.0352025.r001

**Authors:** Hakan Aylanc

31 Mar 2026

PONE-D-25-57012

Mortality amongst European Children with Congenital Anomalies: associations with Socio-economic Status in the EUROLINKCAT cohort

PLOS One

Dear Dr. Jordan,

Thank you for submitting your manuscript to PLOS ONE. After careful consideration, we feel that it has merit but does not fully meet PLOS ONE’s publication criteria as it currently stands. Therefore, we invite you to submit a revised version of the manuscript that addresses the points raised during the review process.

Please submit your revised manuscript by May 15 2026 11:59PM. If you will need more time than this to complete your revisions, please reply to this message or contact the journal office at plosone@plos.org. When you're ready to submit your revision, log on to https://www.editorialmanager.com/pone/ and select the 'Submissions Needing Revision' folder to locate your manuscript file.

Please include the following items when submitting your revised manuscript:

- A letter that responds to each point raised by the academic editor and reviewer(s). You should upload this letter as a separate file labeled 'Response to Reviewers'.
- A marked-up copy of your manuscript that highlights changes made to the original version. You should upload this as a separate file labeled 'Revised Manuscript with Track Changes'.
- An unmarked version of your revised paper without tracked changes. You should upload this as a separate file labeled 'Manuscript'.

If you would like to make changes to your financial disclosure, please include your updated statement in your cover letter. Guidelines for resubmitting your figure files are available below the reviewer comments at the end of this letter.

If applicable, we recommend that you deposit your laboratory protocols in protocols.io to enhance the reproducibility of your results. Protocols.io assigns your protocol its own identifier (DOI) so that it can be cited independently in the future. For instructions see: https://journals.plos.org/plosone/s/submission-guidelines#loc-laboratory-protocols. Additionally, PLOS ONE offers an option for publishing peer-reviewed Lab Protocol articles, which describe protocols hosted on protocols.io. Read more information on sharing protocols at https://plos.org/protocols?utm_medium=editorial-email&utm_source=authorletters&utm_campaign=protocols.

As the corresponding author, your ORCID iD is verified in the submission system and will appear in the published article. PLOS supports the use of ORCID, and we encourage all coauthors to register for an ORCID iD and use it as well. Please encourage your coauthors to verify their ORCID iD within the submission system before final acceptance, as unverified ORCID iDs will not appear in the published article. *Only*  the individual author can complete the verification step; PLOS staff *cannot*  verify ORCID iDs on behalf of authors.

We look forward to receiving your revised manuscript.

Kind regards,

Hakan Aylanc

Academic Editor

PLOS One

Journal Requirements:

When submitting your revision, we need you to address these additional requirements.

1. Please ensure that your manuscript meets PLOS ONE's style requirements, including those for file naming. The PLOS ONE style templates can be found at

https://journals.plos.org/plosone/s/file?id=wjVg/PLOSOne_formatting_sample_main_body.pdf and https://journals.plos.org/plosone/s/file?id=ba62/PLOSOne_formatting_sample_title_authors_affiliations.pdf

2. Your ethics statement should only appear in the Methods section of your manuscript. If your ethics statement is written in any section besides the Methods, please delete it from any other section.

3. We note that you have indicated that there are restrictions to data sharing for this study. PLOS only allows data to be available upon request if there are legal or ethical restrictions on sharing data publicly. For more information on unacceptable data access restrictions, please see http://journals.plos.org/plosone/s/data-availability#loc-unacceptable-data-access-restrictions.

Before we proceed with your manuscript, please address the following prompts:

a) If there are ethical or legal restrictions on sharing a de-identified data set, please explain them in detail (e.g., data contain potentially identifying or sensitive patient information, data are owned by a third-party organization, etc.) and who has imposed them (e.g., a Research Ethics Committee or Institutional Review Board, etc.). Please also provide contact information for a data access committee, ethics committee, or other institutional body to which data requests may be sent.

b) If there are no restrictions, please upload the minimal anonymized data set necessary to replicate your study findings to a stable, public repository and provide us with the relevant URLs, DOIs, or accession numbers. For a list of recommended repositories, please see

https://journals.plos.org/plosone/s/recommended-repositories. You also have the option of uploading the data as Supporting Information files, but we would recommend depositing data directly to a data repository if possible.

We will update your Data Availability statement on your behalf to reflect the information you provide.

4. In the online submission form, you indicated that data were used under license for the current study. Because restrictions apply to the availability of these data, there are no publicly available data. Data can be released from the authors for scientifically valid requests, upon permission of the participating registers of congenital anomalies.

All PLOS journals now require all data underlying the findings described in their manuscript to be freely available to other researchers, either 1. In a public repository, 2. Within the manuscript itself, or 3. Uploaded as supplementary information.

This policy applies to all data except where public deposition would breach compliance with the protocol approved by your research ethics board. If your data cannot be made publicly available for ethical or legal reasons (e.g., public availability would compromise patient privacy), please explain your reasons on resubmission and your exemption request will be escalated for approval.

5. Please amend your authorship list in your manuscript file to include author Lyuba Ostapchuk, Wladimir Wertrlecki.

6. Please amend the manuscript submission data (via Edit Submission) to include author Lyubov Ostapchuk, Wertelecki W.

7. Please amend your list of authors on the manuscript to ensure that each author is linked to an affiliation. Authors’ affiliations should reflect the institution where the work was done (if authors moved subsequently, you can also list the new affiliation stating “current affiliation:….” as necessary).

8. If the reviewer comments include a recommendation to cite specific previously published works, please review and evaluate these publications to determine whether they are relevant and should be cited. There is no requirement to cite these works unless the editor has indicated otherwise.

Additional Editor Comments :

We look forward to receiving your revisions as soon as possible, in response to the reviewers' comments. Best of wishes.

[Note: HTML markup is below. Please do not edit.]

Reviewers' comments:

Reviewer's Responses to Questions

**Comments to the Author**

1. Is the manuscript technically sound, and do the data support the conclusions?

The manuscript must describe a technically sound piece of scientific research with data that supports the conclusions. Experiments must have been conducted rigorously, with appropriate controls, replication, and sample sizes. The conclusions must be drawn appropriately based on the data presented.

Reviewer #1: Partly

**********

2. Has the statistical analysis been performed appropriately and rigorously?

Reviewer #1: Yes

**********

3. Have the authors made all data underlying the findings in their manuscript fully available?

The PLOS Data policy requires authors to make all data underlying the findings described in their manuscript fully available without restriction, with rare exception (please refer to the Data Availability Statement in the manuscript PDF file). The data should be provided as part of the manuscript or its supporting information, or deposited to a public repository. For example, in addition to summary statistics, the data points behind means, medians and variance measures should be available. If there are restrictions on publicly sharing data—e.g. participant privacy or use of data from a third party—those must be specified.

Reviewer #1: No

**********

4. Is the manuscript presented in an intelligible fashion and written in standard English?

PLOS ONE does not copyedit accepted manuscripts, so the language in submitted articles must be clear, correct, and unambiguous. Any typographical or grammatical errors should be corrected at revision, so please note any specific errors here.

Reviewer #1: Yes

**********

5. Review Comments to the Author

Please use the space provided to explain your answers to the questions above. You may also include additional comments for the author, including concerns about dual publication, research ethics, or publication ethics. (Please upload your review as an attachment if it exceeds 20,000 characters)

Reviewer #1: This manuscript investigates the impact of socio-economic status (SES) on mortality among children with major congenital anomalies up to age 10, using data from the EUROlinkCAT cohort. The study addresses a significant gap in the literature by extending the analysis beyond infancy. The use of a large, multi country cohort is a major strength. However, there are significant methodological concerns regarding the heterogeneity of the study population and the lack of a reference group that need to be addressed to validly interpret the findings.

Major Revisions:

1-Stratification by Anomaly Subgroups: The current analysis treats major congenital anomalies as a single, homogenous group; however, from a clinical and genetic perspective, this approach obscures critical differences. The natural history, mortality risk, and dependency on high resource medical interventions vary drastically across diverse categories, ranging from complex congenital heart defects and neural tube defects to chromosomal abnormalities and isolated limb defects. Therefore, the authors should perform a subgroup analysis stratified by major organ systems or broad anomaly categories, such as isolated congenital heart defects, chromosomal anomalies, and non-chromosomal structural defects. Even if sample sizes limit granular analysis, this broad stratification is essential to determine if the SES gradient is driven by specific, resource dependent pathologies.

2-The study analyzes mortality differences within the congenital anomaly cohort but lacks a comparison with the background mortality of children without anomalies. This reference group is necessary to distinguish whether low SES exerts a disproportionately higher negative effect on children with anomalies compared to their healthy peers or merely reflects known background inequalities. The authors should include mortality rates for children without congenital anomalies from the same regions to demonstrate if the socioeconomic mortality gap is significantly wider in the anomaly group.

**********

6. PLOS authors have the option to publish the peer review history of their article (what does this mean?). If published, this will include your full peer review and any attached files.

If you choose “no”, your identity will remain anonymous but your review may still be made public.

**Do you want your identity to be public for this peer review?**  For information about this choice, including consent withdrawal, please see our Privacy Policy.

Reviewer #1: No

**********

[NOTE: If reviewer comments were submitted as an attachment file, they will be attached to this email and accessible via the submission site. Please log into your account, locate the manuscript record, and check for the action link "View Attachments". If this link does not appear, there are no attachment files.]

To ensure your figures meet our technical requirements, please review our figure guidelines: https://journals.plos.org/plosone/s/figures

You may also use PLOS’s free figure tool, NAAS, to help you prepare publication quality figures: https://journals.plos.org/plosone/s/figures#loc-tools-for-figure-preparation.

NAAS will assess whether your figures meet our technical requirements by comparing each figure against our figure specifications.

# Author comment: Author response to Decision Letter 1

**DOI:** 10.1371/journal.pone.0352025.r002

15 May 2026

Reviewer #1: This manuscript investigates the impact of socio-economic status (SES) on mortality among children with major congenital anomalies up to age 10, using data from the EUROlinkCAT cohort. The study addresses a significant gap in the literature by extending the analysis beyond infancy. The use of a large, multi country cohort is a major strength.

Thank you for your support.

However, there are significant methodological concerns regarding the heterogeneity of the study population and the lack of a reference group that need to be addressed to validly interpret the findings.

Major Revisions:

1-Stratification by Anomaly Subgroups: The current analysis treats major congenital anomalies as a single, homogenous group; however, from a clinical and genetic perspective, this approach obscures critical differences. The natural history, mortality risk, and dependency on high resource medical interventions vary drastically across diverse categories, ranging from complex congenital heart defects and neural tube defects to chromosomal abnormalities and isolated limb defects.

Therefore, the authors should perform a subgroup analysis stratified by major organ systems or broad anomaly categories, such as isolated congenital heart defects, chromosomal anomalies, and non-chromosomal structural defects. Even if sample sizes limit granular analysis, this broad stratification is essential to determine if the SES gradient is driven by specific, resource dependent pathologies. We agree, and thank you for this suggestion.

This study is restricted to major congenital anomalies, as defined by EUROCAT: major congenital anomalies are structural changes that have significant medical, surgical, social or cosmetic consequences for the affected individual, and typically require medical intervention. Nevertheless, variation exists within and between diagnostic categories, sometimes depending on the severity of the lesion (for spina bifida this is the level of the lesion). Unfortunately, EUROCAT registers are unable to capture severity, and hence resource demands.

With this caveat, we now tabulate the full range of anomalies, where there are adequate numbers to work within governance approvals, in a supplementary file S3 file. S3, S4 Tables. However, the low number of deaths precluded analysis for many anomalies, as elsewhere (Tan et al 2023). In most countries, CHD and severe CHD had sufficient subjects for analysis, and the results are tabulated separately in S2 file, S2 Table.

We have written under methods, line 187:

Cox proportional hazard ratios in individual congenital anomalies were reported for individual countries where >50 deaths occurred, without meta-analysis (Austin et al 2017).

Under results, line 284 et seq.: The associations between SES and CHD and severe CHD were stronger in childhood than infancy, and, in the UK, for the most deprived groups; this trend was less apparent in Southern Europe, particularly where there were few cases (S2 Table). In infancy, in some countries, hazard ratios were high for spina bifida, hydrocephalus, congenital cataracts, gastroschisis, hydronephrosis, limb reduction, and craniosynostosis, but low numbers preclude interpretation. Deprivation was not associated with infant mortality for children with facial clefts, multicystic renal dysplasia or hypospadias (S3 Table). The low numbers of deaths in children aged 1-10 precluded interpretable calculations. In some registers, associations between CHD, severe microcephaly, limb defects, facial clefts or hydrocephalus and childhood mortality were apparent (S4 Table).

Under discussion, line 352 et seq. :

The anomalies most affected by deprivation (CHD, severe CHD, Down syndrome (infants), limb defects, hydrocephalus, craniosynostosis, severe microcephaly, gastroschisis) were those requiring extensive surgery and rehabilitation. The anomalies least affected by deprivation, (hypospadias, multicystic renal dysplasia, Down syndrome (ages 1-10) often need less aftercare following initial surgery, reducing financial [63] and care demands [79].

2-The study analyzes mortality differences within the congenital anomaly cohort but lacks a comparison with the background mortality of children without anomalies. This reference group is necessary to distinguish whether low SES exerts a disproportionately higher negative effect on children with anomalies compared to their healthy peers or merely reflects known background inequalities. The authors should include mortality rates for children without congenital anomalies from the same regions to demonstrate if the socioeconomic mortality gap is significantly wider in the anomaly group.

Thank you for this suggestion. We agree this is important. We sought population data from the public domain, and the register leads to report infant and childhood mortality according to SES. Where available, this has been added to Table 1. We note that EUROSTAT do not report these data. This accentuates the importance of our data. The sparsity of data limits interpretation, and we acknowledge this. International inconsistency in reporting of SES is an acknowledged limitation.

We have explained under ‘settings’, acknowledging the contribution of congenital anomalies to deaths in infancy and childhood, line 108:

Population infant mortality, and SES gradations in infant and childhood mortality rates were reported to illustrate comparability with whole-population mortality (Table 1); some 20-31% infant and childhood deaths are attributed to congenital anomalies [PHW 2022, Statistics Finland nd, ONS 2025].

We have commented under ‘discussion’, line 319 et seq.:

In Wales, but not England, the impact of SES of infant mortality differed little between infants with anomalies and the whole population. Data for childhood deaths indicate a sharper contrast between rich and poor amongst children with anomalies and the whole population (albeit sometimes based on different age ranges). Similarly, in Finland, the mortality hazard ratios for children aged 1-9 born outside the EU were higher than the rate ratios between high and low SES. Data are too sparse to speculate on the impact of resource constraints [63]

We acknowledge, line 385:

We were unable to report on population infant mortality according to SES for some registers. Data for childhood deaths were limited, with only Finland and Wales able to report deaths for ages 1-9.

________________________________________

Attachment Submitted filename: Response to reviewers.docx

# Editor report: Decision Letter 1

**DOI:** 10.1371/journal.pone.0352025.r003

**Authors:** Hakan Aylanc

4 Jun 2026

Mortality amongst European Children with Congenital Anomalies: associations with Socio-economic Status in the EUROLINKCAT cohort

PONE-D-25-57012R1

Dear Dr. Jordan,

We’re pleased to inform you that your manuscript has been judged scientifically suitable for publication and will be formally accepted for publication once it meets all outstanding technical requirements.

Within one week, you’ll receive an e-mail detailing the required amendments. When these have been addressed, you’ll receive a formal acceptance letter and your manuscript will be scheduled for publication.

An invoice will be generated when your article is formally accepted. Please note, if your institution has a publishing partnership with PLOS and your article meets the relevant criteria, all or part of your publication costs will be covered. Please make sure your user information is up-to-date by logging into Editorial Manager at Editorial Manager® and clicking the ‘Update My Information' link at the top of the page. For questions related to billing, please contact billing support.

If your institution or institutions have a press office, please notify them about your upcoming paper to help maximize its impact. If they’ll be preparing press materials, please inform our press team as soon as possible -- no later than 48 hours after receiving the formal acceptance. Your manuscript will remain under strict press embargo until 2 pm Eastern Time on the date of publication. For more information, please contact onepress@plos.org.

Kind regards,

Hakan Aylanc

Academic Editor

PLOS One

Additional Editor Comments (optional):

Reviewers' comments:

# Editor report: Acceptance letter

**DOI:** 10.1371/journal.pone.0352025.r004

**Authors:** Hakan Aylanc

PONE-D-25-57012R1

PLOS One

Dear Dr. Jordan,

I'm pleased to inform you that your manuscript has been deemed suitable for publication in PLOS One. Congratulations! Your manuscript is now being handed over to our production team.

At this stage, our production department will prepare your paper for publication. This includes ensuring the following:

* All references, tables, and figures are properly cited

* All relevant supporting information is included in the manuscript submission,

* There are no issues that prevent the paper from being properly typeset

You will receive further instructions from the production team, including instructions on how to review your proof when it is ready. Please keep in mind that we are working through a large volume of accepted articles, so please give us a few days to review your paper and let you know the next and final steps.

Lastly, if your institution or institutions have a press office, please let them know about your upcoming paper now to help maximize its impact. If they'll be preparing press materials, please inform our press team within the next 48 hours. Your manuscript will remain under strict press embargo until 2 pm Eastern Time on the date of publication. For more information, please contact onepress@plos.org.

You will receive an invoice from PLOS for your publication fee after your manuscript has reached the completed accept phase. If you receive an email requesting payment before acceptance or for any other service, this may be a phishing scheme. Learn how to identify phishing emails and protect your accounts at https://explore.plos.org/phishing.

If we can help with anything else, please email us at customercare@plos.org.

Thank you for submitting your work to PLOS ONE and supporting open access.

Kind regards,

PLOS ONE Editorial Office Staff

on behalf of

Dr. Hakan Aylanc

Academic Editor

PLOS One
